# Increasing the stability margins using multi-pattern metasails and multi-modal laser beams

**DOI:** 10.1038/s41598-022-24681-w

**Published:** 2022-11-21

**Authors:** Mohammadrasoul Taghavi, Hossein Mosallaei

**Affiliations:** Metamaterials Laboratory Electrical and Computer Engineering Department, Northeastern University, Boston, MA 02115 USA

**Keywords:** Aerospace engineering, Electrical and electronic engineering

## Abstract

Laser-driven metasails can enable reaching velocities far beyond the chemically propelled spacecrafts, which accounts for precise engineering of the acceleration and the stability degree of the lightsail across the Doppler-broadened band. All-dielectric metasurfaces have shown great promise toward the realization of low-weight photonic platforms suitable for integrating multiple functionalities. The most paramount factor in the stability analysis of lightsail is the coupling between displacement and rotation, which mainly determines the durability of the nanocraft against displacement and rotation offsets. In this work, the marginal stability conditions of laser-propelled lightsails have been extended by replacing the reflective elements near the edges portions of the sail with broad-band transmissive elements and applying a multi-objective genetic algorithm (GA) optimization to the proposed configuration. The presented design not only remarkably suppresses the amplitude of the oscillatory motion but also can decrease the center of the mass requirement of the lightsail while maintaining an acceptable acceleration time. Next, a configuration where the payload is at the non-illuminating side of the dual-portion sail is proposed to protect the payload from the intense laser beam. In this case, a spherical phase profile is imprinted across the reflective elements while it is being propelled by a multi-modal beam.

## Introduction

**Figure 1:**
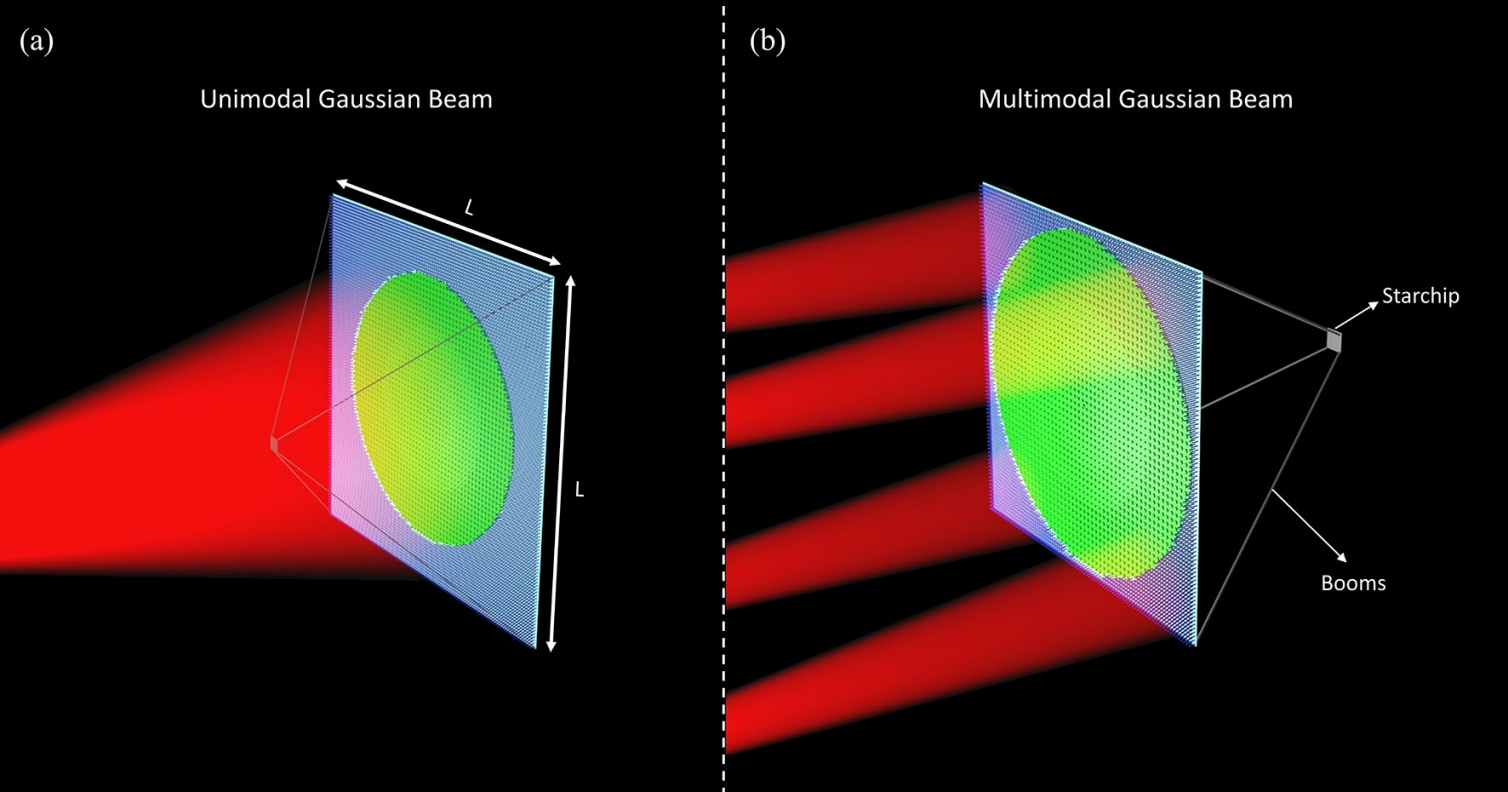
(**a**) Schematic depiction of nanocraft consisting of a dual-portion metasail and a payload at the illumination side, deployed in the outer space being accelerated by a uni-modal propulsion beam from an Earth-based laser array. (**b**) Similar configuration with the payload placed at the back-side for protecting it from the high-power laser beams, being accelerated by a multi-modal laser beam. In both of the cases we have assumed the dimension of the sail as $L = 2$ m and the net power of the beams as $P_0 = 100$ GW. The central and outer portions of the metasail consists of a reflective and transmissive c-Si nanodisks over a silica substrate and there exists a thin layer of c-Si between the reflective elements and the substrate. Generated by Autodesk 3ds Max.

Using the momentum of light, a propellant has initiated a new generation of gram-scale nanocrafts that can reach relativistic speeds within a few minutes of propulsion^1–9^. More precisely, Breakthrough Starshot is an initiative aiming to send a swarm of nanoscale lightsails to probe Proxima Centauri B, an exoplanet orbiting in the habitable zone of the closest star system, by a flyby mission using an Earth-based high-power laser arrays^10^. Originally, optical forces have been employed for various applications in the area of microscopic nanoparticle manipulation,trapping and acceleration and thin membrane pressurizing^11–16^. The promising utilization of optical forces for mechanical manipulation of nanoscale objects has sparked a great interest in harnessing the radiation pressure of light for accelerating large-scale and lightweight spacecrafts namely, solarsails and lightsails^17–19^. Lightsailing technology is an emerging area that encounters different branches of science and engineering for designing nanocrafts for extended mission applications. Although the realization of such a mission does not violate any physical laws, it demands considerable improvements in the area of sail, Starchip, and laser array designing. Moreover, one of the crucial requirements for successful photonic propulsion is an ultimate design of lightsail to accomplish a stable beam-riding while minimizing acceleration time along with proper thermal management via radiative cooling^3,6,20,21^. Successful beam-riding requires the generation of sufficient restoring force and torques against the possible displacement and tilt of the beam with respect to the center of the sail^1,2,22–28^. Previously, it has been well understood that flat macroscopic structures are unequivocally unstable and any slight misalignment or displacement will cause the sail to deviate away from the center of the beam immediately. In order to introduce a self-stabilizing mechanism in the spacecraft, spherical, conical, and rigid parabolic structures were studied, and it has been proven that under the specific configurations, conical and parabolic structures can maintain in the beam area with oscillatory motions during the acceleration period^2,23^. However, challenges related to the material selection and conserving their initial rigid structure have made their realization very demanding. Recently photonic metasurfaces have shown a great promise toward realization of relativistic lightweight nanocrafts thanks to their unparalleled domination over the magnitude and direction of the reflected and transmitted beam’s wavefront^5–8,29,30^. Moreover, photonic metasurfaces enable anomalous reflection and transmission of the incident light by creating spatially variant phase discontinuity over the aperture of the sail, giving rise to prices government over the in-plane forces which is of paramount importance for designing self-stabilizing metasails. Reaching relativistic velocities calls for a gram-scale total mass of the spacecraft while retaining maximal control over the mechanical performance of the sail, which can be achieved by all-dielectric photonic metasails due to their unique properties such as ultra-thin and low-density structure. The flat all-dielectric photonic metasail can imitate the optical performance of an ideal macroscopic structure while satisfying the mass and thermal management requirements of the relativistic spacecraft, which are keys to a successful launch of lightsails. Nevertheless, all-dielectric photonic platforms hold great promise in bringing advanced multifunctional performances for extended mission applications that are not viable with other platforms. Recently there has been experimental demonstration of the beam-riding and material characterization of the lightsails which can pave the way toward realization of relativistic lightsails^31–34^. In this work, we have proposed a novel structure for extending the marginal stability basins and reducing the center of mass distance requirements of the metasail by applying a multi-objective optimization to the dual-pattern photonic metasail for the different center of mass distances. The exerted optical forces and torques are derived in this scenario, followed by a motion trajectory study to highlight the remarkable effect of the implemented technique on increasing the stability degree of the sail while minimizing the reduction in thrust as a result of substituting the reflective elements with transmissive ones. Moreover, we have investigated the possibility of deploying the Starchip at the back (non-illuminating side) of the lightsail and the required propulsion beam shape to ensure stable beam-riding performance. It should be emphasized that this type of arrangement of the sail brings several advantages including protection of the Starchip against the intense laser beam and the heat caused by it and also providing a new degree of freedom in designing dynamic and time-varying beams that can pave the way for realization of such nanoscale spacecrafts which can be further studied. As shown in Fig. 1, the proposed nanocraft consists of an all-dielectric dual-pattern lightsail $2\times 2\;\text {m}^{2}$ connected to the Starchip in a parachute configuration via rigid booms. Depending on the operation principle, the Starchip is placed in the front and back side of the sail, which necessitates single and multi-modal Gaussian beam profiles, respectively. It should be noted that both the single and multi-modal Gaussian beams are considered to be unpolarized with total power of 100 GW, distributed equally between the perpendicular (s-) and parallel (p-) polarizations, to account for polarization alteration during their path till arriving at the lightsail. Throughout the paper, we perform a relatively simplified multi-physics simulation to combine the optical and mechanical characteristics of the lightsail to obtain the dynamics of the sail during the acceleration to achieve an extensive understanding of the role of the introduction of a transmissive nanoscale pattern in expanding the stability margins of the light sail. Also, we study the effect of positioning the center of mass at the back side of the sail on the sail’s transverse dynamics. In both scenarios, we estimate the motion trajectory during the acceleration stage.

The rest of the manuscript is organized as follows. In “Nanoscale photonic unitcell design”, we outline the design procedure of the dual-pattern photonic metasurface and investigate its performance in the propulsion band. In “Optomechanical analysis framework”, we briefly discuss the optomechanical analysis framework that is adopted for analyzing the problem. The multi-objective optimization-based design of dual-pattern metasail and its motion trajectory performance are discussed in “Dual-pattern metasail with uni-modal beam”. In “Dual-pattern metasail with multi-modal beams”, a novel configuration of nanocraft in which the Starchip is placed at the back-side of the sail is proposed and its motion trajectory is also studied. Finally, the conclusion is given in “Conclusion”.

## Nanoscale photonic unitcell design

All-dielectric metasurfaces are great candidates for accommodating the laser propulsion requirements of the relativistic nanocrafts owing to their complex wavefront control ability and low-weight structure^35–37^. Furthermore, the first step in designing such relativistic nanocrafts is to properly outline the stable beam-riding requirements by studying the propulsion stage. According to the atmospheric transparency window choosing the ground-based laser wavelength as 1.3 $\upmu$m seems ideal, resulting in a Doppler-broadened band of 1.3 $\upmu$m–1.55 $\upmu$m^3^. The proposed designs will consist of two different unitcells, reflective and transmissive, with high overall spatial reflectivity and transmissivity in the Doppler-broadened propulsion spectrum, respectively. Nevertheless, both unitcells are designed to provide a wide phase span throughout the Doppler-broadened propulsion band. Based on the application, all-dielectric metasurfaces can operate both in reflective and transmissive regimes by offering a broad phase accumulation^37–43^. Moreover, sub-wavelength dielectric resonators enable the excitation of electric dipole (ED) and magnetic dipole (MD) resonances which can operate in the reflection with high efficiencies^38^. However, operating in either electric dipole (ED) or magnetic dipole (MD) does not provide a wide phase span for broadband spectrum applications. Among the available methods for rendering broadband reflecting metasurfaces, adopting a design based on the combination of guided-mode resonance (GMR) and magnetic dipole resonance (MD) seems to be ideal, which is enabled by adding a subwavelength dielectric layer leading to expansion of the reflection bandwidth and phase span^6,44–46^. As outlined in the previous section of the manuscript, in addition to the broadband reflective metasurface, a broadband transmissive metasurface in the Doppler-broadened spectrum is also deemed. Generally, transmissive metasurfaces can be achieved by spectrally overlapping the electric dipole (ED) and the magnetic dipole (MD) resonances of the all-dielectric nanoresonators to satisfy the Huygens’ condition. However, these kinds of designs suffer from low efficiency due to interelement field coupling, which makes them unsuitable for complex beam steering applications^47^. Using high aspect ratio nanopillars is shown to provide a viable solution toward realizing broadband all-dielectric metasurfaces with high efficiencies^47–50^. It should be noted that all of the simulations are done using MATLAB and the schematics are generated using Autodesk 3ds Max^51,52^. The tall nanodisks with high heights $h \sim \lambda$ can support hybrid Mie–Fabry–Perot (HM-FP) resonances, yielding approximately $2\pi$ phase span over a broad spectrum.

**Figure 2:**
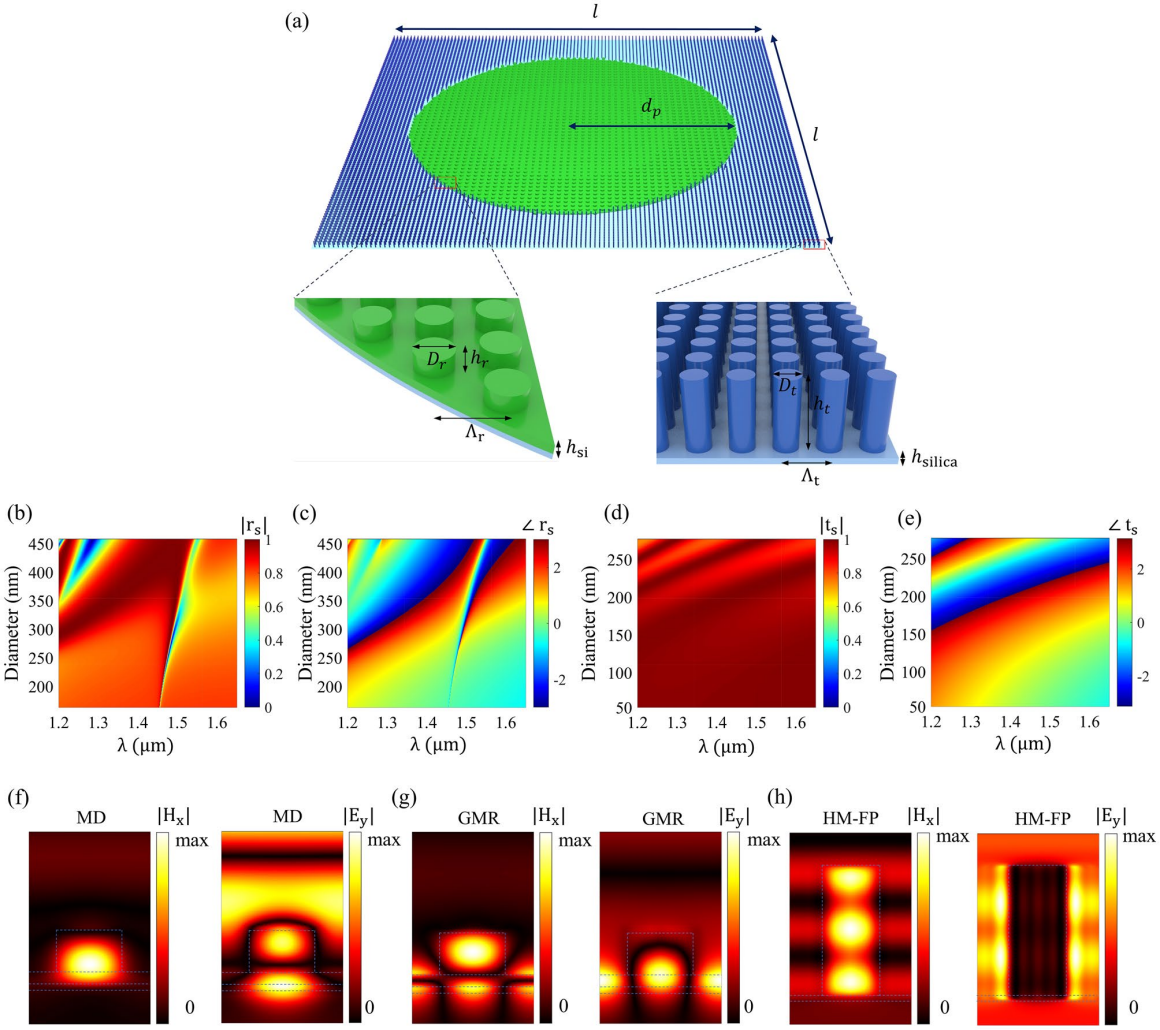
(**a**) The geometry of all-dielectric $2\times 2\;\text {m}^2$ metasurface consisting of broadband reflective and transmissive unitcells. The reflective c-Si nanodisks are connected by a thin c-Si layer over a silica substrate while the tall transmissive elements are placed over the silica substrate and the parameter $d_p$ determines the reflective portion radius. The dimension of the building blocks are given as: $h_r\;=\;306\;\text {nm}$, $\Lambda _r\;=\;653\;\text {nm}$, $h_{\text {Si}}\;=\;103\;\text {nm}$, $h_{\text {silica}}\;=\;60\;\text {nm}$ and $163\;\text {nm}<D_r< 458\;\text {nm}$ and $h_t\;=\;1349\;\text {nm}$, $\Lambda _t\;=\;323\;\text {nm}$, $h_{\text {silica}}\;=\;60\;\text {nm}$ and $54\;\text {nm}<D_t< 277\;\text {nm}$. The reflection amplitude (**b**) and phase (**c**) of the reflective unitcells under illumination of a s-polarized plane wave. The transmission amplitude (**d**) and phase (**e**) of the transmissive unitcells under illumination of a s-polarized planewave. (**f**,**g**) The nearfield distribution of the magnetic and electric fields in the $y-z$ plane at the resonant wavelengths of MD and GMR for s-polarized normal incidence, respectively. (**h**) Nearfield distribution of the magnetic and electric fields of transmissive unitcells under normal s-polarized incidence demonstrating the Hybrid Mie–Fabry–Perot (HM-FP) resonance. The schematic and results are Generated by Autodesk 3ds Max and MATLAB, respectively.

Among the limited number of allowed materials to be used in the lightsail, we have chosen the Si-silica platform due to its prosperous properties such as ultra-low mass density and thermal durability against extreme temperatures^3^. In order to design a polarization-insensitive reflective metasurface, we have selected c-Si nanodisks over ultra-thin c-Si and silica layers. Our simulation has been conducted within frameworks of the rigorous coupled wave analysis (RCWA) by illuminating the metasurface by plane waves with perpendicular (s-) and parallel (p-) polarization. Also, the experimentally measured refractive indices of c-Si and silica in the near-infrared are used^53^. It is envisioned that the temperature escalation of the sail as a result of a high-power incident beam may alter the optical properties and thermal emissivity of the utilized materials, which should be taken into account however, experimental data for such characterization does not exist. The two different goals of having high reflectivity and maximum phase span call for adopting a multi-objective genetic algorithm (GA) optimization to attain a near-optimal trade-off between the desired performance metrics. Next, the optimized dimensions of the reflective metasurface are the following: $h_r\;=\;306\;\text {nm}$, $\Lambda _r\;=\;653\;\text {nm}$, $h_{\text {Si}}\;=\;103\;\text {nm}$, $h_{\text {silica}}\;=\;60\;\text {nm}$ and $D_r$ varying in the range of 163–458 nm. Similar multi-objective optimization has been employed for designing broadband and wide phase span transmissive metasurfaces. In this case, the objective functions are the transmission amplitude and phase accumulation of the nanoscale unitcell. The optimized dimensions are given as: $h_t\;=\;1349\;\text {nm}$, $\Lambda _t\;=\;323\;\text {nm}$, $h_{\text {silica}}\;=\;60\;\text {nm}$ and $D_t$ varying in the range of 54–277 nm. Figure 2a depicts the dual-pattern metasurface in which the reflective (zero-contrast) and transmissive unitcells are placed in the central and outer regions of the nanoscale structure, respectively. Figure 2b,c shows the reflection and phase amplitudes of the nano building blocks of the reflective metasurface for an s-polarized incident beam, respectively, wherein the magnetic dipole (MD) and guided mode resonances (GMR) are observable. As it is demonstrated, the reflective unitcell possesses relatively high reflectivity with a wide phase span across the propulsion spectrum. It should be noted that the narrow line with low values of reflectivity and the corresponding discontinuity in the phase response in the vicinity of the guided mode resonance branch is due to the destructive interference between the resonant and non-resonant pathways in the transmitted and reflective beams. We will discuss its adverse effects on the motion trajectory performance further^54^. Figure 2d,e illustrate the transmission amplitude and phase of the transmissive metasurface elements. As it can be observed, a very high transmission amplitude with a near $2\pi$ phase span across the propulsion spectrum has been obtained. It is envisioned that the higher order mode interference supported by Hybrid Mie-Fabry-Perot resonances can provide higher degrees of freedom in the static metasurface design^47^. In order to gain a clear picture of the obtained resonances, we have plotted the nearfield distribution of the magnetic and electric nearfields in the vicinity of the MD, GMR, and the hybrid Mie–Fabry–Perot resonances in the $y-z$ plane in Fig. 2f–h, respectively. As can be observed from Fig. 2f, the magnetic field is confined inside the nanodisk, and two antinodes are formed in the electric field distribution along the longitudinal direction which denotes an MD resonance. Figure 2g illustrates the formation of two counter-propagating leaky guided waves, which has led to the appearance of a standing wave pattern inside the matched silicon layer, characterizing a GMR resonance. Also, Fig. 2h depicts the magnetic and electric field distribution along the tall transmissive unitcell supporting hybrid Mie–Fabry–Perot resonance. Nevertheless, the selected value for the height of the silica substrate ensures sufficient thermal emissivity in the Doppler-broadened spectrum^6^. It should be noted that due to the rotational symmetry of the designed reflective and transmissive building blocks, similar results can be obtained for the p-polarized normal incident beam. Another important factor in photonic metasail design is the angular sensitivity of the unitcell’s response, as the sail will experience oscillatory motion resulting in an oblique incidence of the propulsion beam. Further information on the oblique incidence’s effect on the building blocks’ response and, consequently, the performance of the lightsail is brought in “Optomechanical analysis framework” of the supporting information. However, as we will discuss in the next section, for enabling the linear stability analysis, the amplitude of the rotation of the sail is limited to small degrees; hence the change in the response of the reflective and transmissive unitcells is assumed to be negligible^6^. It should be noted that the absence of vibrational modes of silicon in the mid-infrared range makes its emissivity insufficient for the thermal survival of the nanocraft in the propulsion stage. Hence, adding a thin silica layer with high emissivity in the same spectrum (unlike silicon) can help reduce the heterostructure’s steady-state temperature during the acceleration stage^1^. It has been shown that by the addition of the silica layer, the maximal steady-state temperature of the proposed relativistic platform can be kept under its melting point^6^.

To account for imperfections in the fabrication process and their adverse effect on the response of the proposed all-dielectric building blocks, we have performed a tolerance analysis in section S3 of the supporting material. Non-uniform laser beam generates spatially varying temperature distribution across the metasail, which also directly relates to the absorption of the all-dielectric unitcells. Although the dielectric elements of the sail are considered to possess an insignificant amount of loss ($10^{-8}$) in the propulsion band, absorbed power as a result of exposure to the high-power ($100\;\text {GW}$) beam can escalate the absorbed power dramatically. Also, the addition of loss can alter the optical response of the unitcells (especially high-quality factor resonances), which should be considered. More information on the effect of considering a larger amount of loss in the dielectric properties of unitcells on the optomechanical performance of the sail can be found in section S6 of the supporting material.

## Optomechanical analysis framework

In this section, we briefly delineate the analysis platform that is used to evaluate the optomechanical functionality of the proposed nanocraft throughout the Doppler-broadened propulsion band. Further details are provided in the section S1 of the Supporting Information.

**Figure 3:**
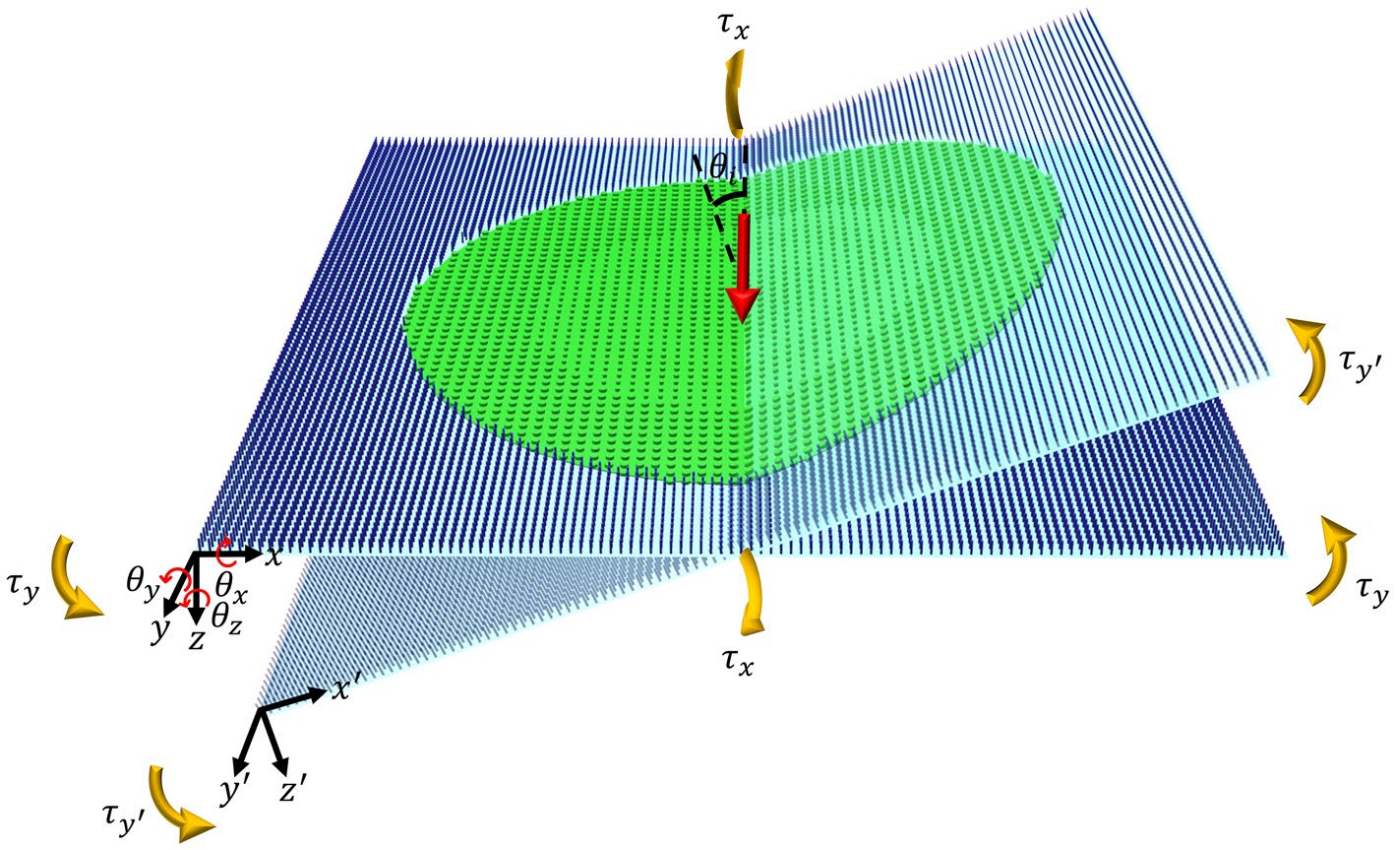
The optomechanical analysis framework for calculating the imparted optical forces in the local coordinate system (primed) and transferring it to the global coordinate system, enabling the linear stability analysis and estimation of motion trajectory in the propulsion period. Generated by Autodesk 3ds Max.

The centerpiece of the analysis method is calculating the imparted optical forces to the sail’s surface under the illumination of an unpolarized high-power laser beam using Maxwell’s stress tensor method (MST)^55^. It should be noted that the large-scale structure of the lightsail consists of trillions of nanoscale building blocks which prohibits employing full-wave simulations; hence we embrace a simplified method centered on local periodicity assumption^6–8^. In this method, the reflection amplitude and phase of the periodic structure of unitcells are obtained using the rigorous coupled wave analysis (RCWA), and then the scattering performance of the macroscopic configuration of the nanoscale elements is predicted by the Generalized Snell’s law, which dwindles the computational complexities considerably^56^. It should be noted that the violation of the local periodicity, mainly for corner elements, and structural imperfections in the large-scale pattern of unitcells will result in spurious and parasitic scattering responses. However, considering their small number with respect to the total number of unitcells, the method is believed to offer sufficiently accurate results for unveiling the essential physics of the problem^57–59^. Assuming the sail, payload, and connecting booms as a rigid body, the dynamics of the nanocraft can be described by Newton Euler’s equations. Figure 3 depicts the dynamic modeling of the metasail in which the local and global coordinate systems of the problem are denoted. Moreover, as the sail preserves an azimuthal symmetry, there would be no in-plane rotating forces ($\tau _z\;=\;0$). Hence the Newton Euler’s equations for slight deviation angles where the following assumption of $\omega _{x,y,z}\;\approx \;\frac{\partial \theta _{x,y,z}}{\partial t}$ holds, can be expressed as a non-linear system of ordinary equations as^60^:

1 $$\begin{aligned} \begin{bmatrix} m_{\text {tot}}\bar{\bar{I}}_{3\times 3} &{} 0 \\ 0 &{} I_{\text {CM}}\bar{\bar{I}}_{2\times 2} \end{bmatrix} \frac{d^2}{dt^2} \begin{bmatrix} x \\ y \\ z \\ \theta _x \\ \theta _y \end{bmatrix} = \begin{bmatrix} F_x \\ F_y \\ F_z \\ \tau _x \\ \tau _y \end{bmatrix} (\textbf{r},\theta _{xy},\nu _z) \end{aligned}$$

Where $I_\text {CM}$ is the principal moment of inertia about the center of mass and assuming the mass of the sail and Starchip equal, it is given by $I_{\text {CM}}=m_{\text {tot}}d^2_{\text {CM}}$. Also, as observable, the forces and torques depend on the position and orientation vectors of the center of mass in the stationary global coordinate system. The velocity ($\nu _z$) of the nanocraft along the incident beam determines the Doppler-sifted wavelength of the received propulsion laser beam ($\lambda (\nu _z)=\lambda _0\sqrt{(1+\nu _z/c)/(1-\nu _z/c)}$)^61^. It should be remarked that the imparted forces and torques possess a nontrivial dependency on the position and rotation of the incident laser beam, which is taken into account by adding an offset and altering the beam’s incident angle. The calculated parameters in the local coordinate system are transformed into the global coordinate system via a set of coordinate transformations to numerically solve the nonlinear system of equations using the Runge-Kutta method. A deeper insight into the stability conditions of the spacecraft can be achieved by projecting the dynamics of the sail to the transverse plane , which renders a 4D oscillator matrix, and obtaining its eigenvalues as^2,62^:$\lambda _1=\lambda _2=0.5(k_1-k_4+\sqrt{(k_1-k_4)^2+4k_1k_4+4k_2k_3})$ and $\lambda _3=\lambda _4=0.5(k_1-k_4-\sqrt{(k_1-k_4)^2+4k_1k_4+4k_2k_3})$ wherein $k_{1,2,3,4}$ are the derivatives of the forces and torques concerning the displacement and rotations in the transverse plane give by:

2 $$\begin{aligned}{} & {} k_1=-\frac{1}{m}\frac{\partial F_x}{\partial x}=-\frac{1}{m}\frac{\partial F_y}{\partial y} \end{aligned}$$

3 $$\begin{aligned}{} & {} k_2=\frac{1}{m}\frac{\partial F_x}{\partial \theta _y}=-\frac{1}{m}\frac{\partial F_y}{\partial \theta _x} \end{aligned}$$

4 $$\begin{aligned}{} & {} k_3=-\frac{1}{I_{\text {CM}}}\frac{\partial \tau _x}{\partial y}=\frac{1}{I_{\text {CM}}}\frac{\partial \tau _y}{\partial x} \end{aligned}$$

5 $$\begin{aligned}{} & {} k_4=\frac{1}{I_{\text {CM}}}\frac{\partial \tau _x}{\partial \theta _x}=\frac{1}{I_{\text {CM}}}\frac{\partial \tau _y}{\partial \theta _y} \end{aligned}$$

Furthermore, it has been demonstrated that the necessary and sufficient condition for marginal stability of the lightsail necessitates the real part of the eigenvalues to be positive, which simplifies to the following equation:$k_1k_4+k_2k_3<0$^2^. In the next sections, two different configurations for the sail’s propulsion scenario are presented based on the imprinted phase gradient and incident laser beam profile. It should be noted that a full-wave RCWA simulation of reflective and transmissive supercells consisting of twelve unitcells for verifying the broad-band functionality and adopted optomechanical method is conducted in the section S3 of the Supporting Information.

## Dual-pattern metasail with uni-modal beam

In this section, we study a $2\times 2\;\text {m}^2$ sail configuration that consists of portions of reflective and transmissive metasurfaces which is illuminated by a uni-modal Gaussian beam with a total power of $P_0\;=\;100\;\text {GW}$ in which the total power is distributed equally between the s and p polarization. Moreover, the equation describing the uni-modal incident laser beam is given:

6 $$\begin{aligned} E(x,y)=\frac{2}{w_0}\sqrt{\frac{\eta P_0}{\pi }}\exp (\frac{-x^2-y^2}{w_0^2}) \end{aligned}$$

where $w_0$ and $P_0$ are the spot size and the total power of the beam when it arrives at the aperture of the sail, respectively, and $\eta$ is the free-space impedance. By choosing the beam profile as a simple Gaussian, the task is obtaining a suitable phase profile to be used as a means to enable a passive self-stabilizing mechanism during the acceleration. It has been successfully demonstrated that defining a parabolic phase gradient which is given by $\varphi (x,y)=-\frac{2\pi }{\lambda _0}(\sqrt{x^2+y^2+F_{\text {p}}}-F_{\text {p}})$ where $F_\text {p}$ is the focal distance of the parabola, over the aperture of the sail consisting of only reflective elements allows a stable beam-riding with residual motions in the terminal velocity^6,8^. Nevertheless, minimizing the amplitude of the sail’s oscillatory motion is crucial for preventing the sail from losing its path en route to its destination. Generally, metasails with parabolic or conical phase profiles under the incidence of a single Gaussian beam satisfy the marginal stability condition as this kind of arrangement yields $k_1>0$ and $k_4<0$, which represents the restoring lateral forces and counterbalancing torques, respectively. Moreover, when the sail is displaced in the transverse plane, the lightsail exploits its parabolic profile to point the reflected beam toward its center of mass, generating a restoring force that brings the sail into its initial condition. It should be emphasized that the most critical parameter in the stability analysis of the lightsail is the coupling between the displacement and the rotation of the sail. The ratio of the following inequality expresses the mentioned coupling $|k_2k_3|<<|k_1k_4|$, which is assumed to decrease the stability basins of the sail noticeably. In the design of a lightsail, there is a trade-off between the acceleration and the amount of imparted restoring forces and torques, which is governed by the scattering response of nanostructured patterns, focal distance, and center of mass of the metasail. Here, we have replaced the reflective metasurfaces at the outer portion of the sail with the transmissive ones to minimize the displacement and rotation cross-coupling. Successful photonic propulsion necessitates maximization of the acceleration and stability margins simultaneously. Increasing the self-stabilizing performance requires enlargement of in-plane lateral forces, which in turn lowers the longitudinal driving force and hence the acceleration time grows. As the acceleration is proportional to the thrust in the nanocraft and the total mass variation is insignificant for different values of $d_p$, we will consider the thrust as the optimization parameter for simulations.

**Figure 4:**
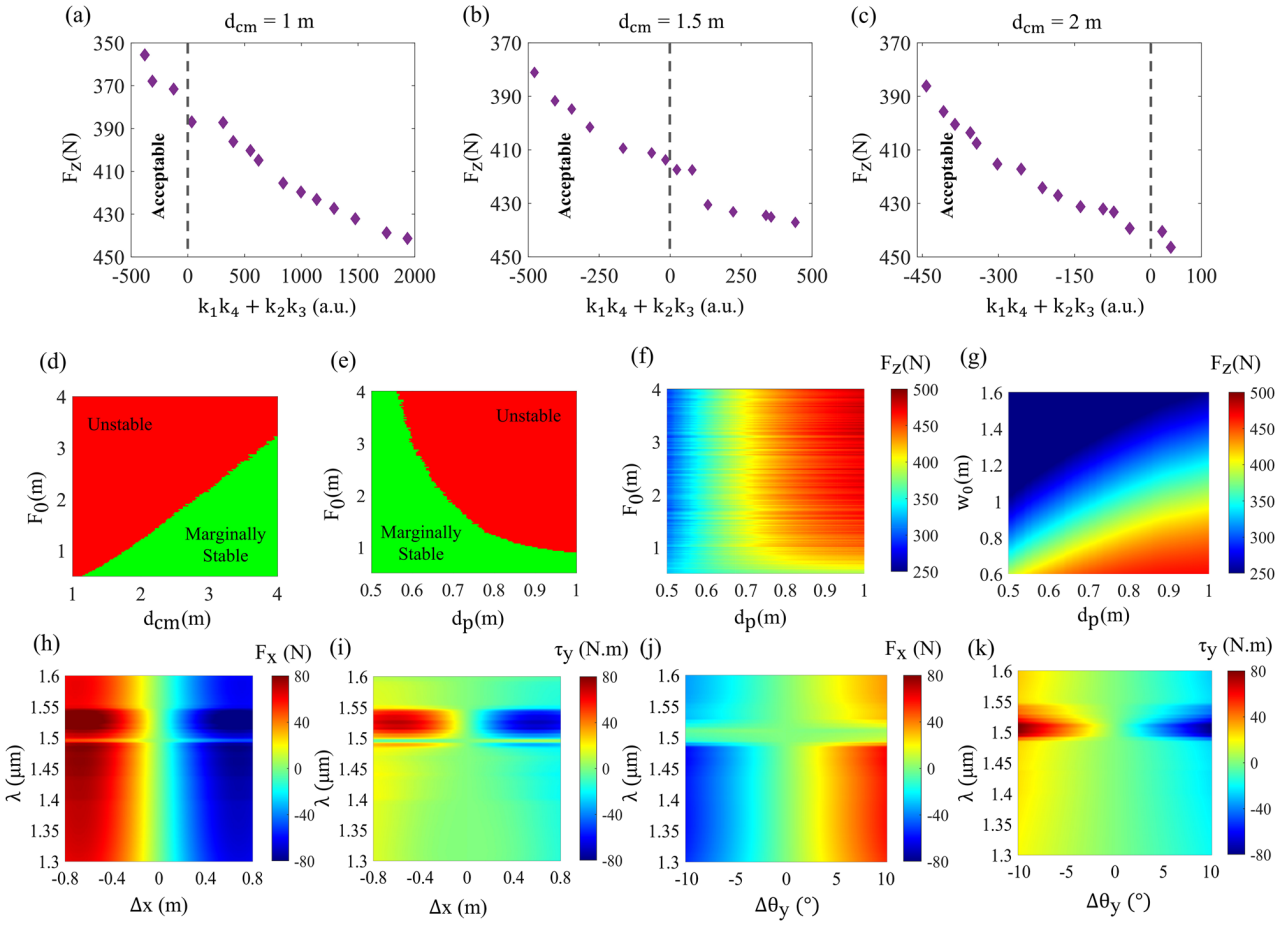
(**a**–**c**) depicts the obtained Pareto-fronts from the multi-objective optimizations for the center of mass distances of $d_{\text {CM}}\;=\;1\;m$ , $d_{\text {CM}}\;=\;1.5\;\text {m}$ and $d_{\text {CM}}\;=\;2\;\text {m}$ , respectively. Among the designs in the solution-space denoted by the Pareto-fronts, the acceptable solutions must satisfy the stability condition $k_1k_4+k_2k_3<0$. (**d**) The marginal stability condition as a function of focal distance of the imprinted phase gradient and the center of mass distance of the spacecraft with $d_p = 0.8$ m. (**e**,**f**) The marginal stability condition and imparted net thrust as a function of focal distance and radius of central reflective portion of the lightsail for the center of mass located at $d_{\text {CM}}\;=\;2\;\text {m}$. It should be noted that $d_p$ is the radius of reflective portion. (**g**) The imparted net thrust as a function of beam spot size and radius of central reflective portion. (**h**,**i**) The calculated $F_x$ and $\tau _y$ components of the imparted in-plane force and torque as a function of displacement along the x-axis. (**j**,**k**) The same, as a function of rotational off-set around the y-axis. Generated by MATLAB.

Furthermore, acceleration time is of crucial importance in realization of the relativistic spacecrafts as it has a direct impact on the required power and size of the ground laser arrays to keep the beam diffraction-limited using the adaptive optics method^63–68^. In order to achieve a near-optimal trade-off between the stability and thrust, an evolutionary multi-objective optimization based on genetic algorithm (GA) namely controlled elitist non-dominated sorting genetic algorithm (CE-NSGA) which is implemented in MATLAB’s Global Optimization Toolbox is adopted^51,69,70^. Moreover, by choosing a simple Gaussian beam with a spot size of $w_0\;=\;0.8\;\text {m}$, the radius of the reflective metasurface can be optimized to obtain a subset of solutions for having a near-optimal trade-off between the stability degree and Thrust. The cost functions for the multi-objective optimization problem can be written as:

7 $$\begin{aligned}{} & {} \underset{P}{maximize}\;\;\;\;\{\text {acceleration}(\lambda )\}(P) \end{aligned}$$

8 $$\begin{aligned}{} & {} \underset{P}{minimize}\;\;\;\;\{k_1(\lambda )k_4(\lambda )+k_2(\lambda )k_3(\lambda )\}(P) \end{aligned}$$

where the $\lambda \;=\;1.35\;\mu \text {m}$ denotes the chosen wavelength for performing the multi-objective optimization. It is noteworthy that we have considered the focal distances of the reflective and transmissive metasurfaces equal to prevent misalignment in the direction of the in-plane transverse forces due to their detrimental effect on the motion trajectory of the sail. Figure 4a–c show the obtained Pareto-front for three different centers of mass distances on the illumination side, wherein the horizontal and vertical axis denotes the stability condition and thrust, respectively. The acceptable zone for selecting a solution in all cases is where the marginal stability condition is satisfied $k_1k_4+k_2k_3<0$. The trade-off clearly illustrates that increasing the stability margins comes at the expense of decreasing the thrust and hence the acceleration. It can be observed that the center of mass distance of the metasail does not affect the thrust value; however, it acts as a biasing parameter to control the imparted restoring torques. Nevertheless, the center of mass distance impacts the amplitude of the residual motion and rotation of the metasail due to changing the magnitude of cross-coupling between the displacement and rotation. It should be noted that marginal stability analysis does not guarantee the stability of the real non-linear system. To mark a design as stable, a complete motion trajectory analysis is necessary. In order to evaluate the solutions on the Pareto-front, one of the designs in the case where the center of mass distance is located two meters away from the sail on the illumination side, which corresponds to $d_p\;=\;0.8\;m$ and $F_p\;=\;0.9\;m$ is selected. In order to get an insight into the effect of the interplay between focal distance and center of mass distances of the reflective and transmissive metasurfaces, we have conducted a stability analysis, and the corresponding result is shown in Fig. 4d. According to Fig. 4d, it is clear that adding a transmissive portion to the sails area has decreased the minimum required center of mass distance for an arbitrarily chosen focal distance from $min\;d_{\text {CM}}\;=\;4\times F_p$ to $min\;d_{\text {CM}}\;=\;2\times F_p$ concerning the previous works^6,8^. In addition, Fig. 4e depicts the effect of changing the radius of the reflective metasurface $d_p$ and focal distance of the metasurfaces for the center of mass distance $d_{\text {CM}}\;=\;2\;$m. It clearly shows that decreasing the transmissive metasurface portion decreases the stability of the lightsail as the cross-coupling between the displacement and rotation escalates. The obtained result verifies the effectiveness of the implemented method for tackling the stability issue in the photonic accelerated nanocrafts as the marginal stability degree boosts by decreasing reflective portions’ diameter $d_p$. In addition, Fig. 4f shows the amplitude of net thrust for various radii of reflective metasurface portions and focal distances. As it can be observed, by adding a transmissive portion, the number of net thrusts decreases for any metasurface focal distance; however, the amount of the change in the thrust is tolerable considering the amount of stability improvement it offers. Also, according to Fig. 4f, increasing the focal distance boosts the net thrust, which comes at the cost of diminished stability conditions. Figure 4g shows the calculated net thrust for different beam spot size $w_0$ and reflective metasurface radial distances in which the net thrust decreases with widening the beam spot size $w_0$. It is concluded from the results that the stability degree of the sail has a direct relationship with the radius of the reflective metasurface radius $d_p$, as by decreasing it, the stability margins widen. The maximum net thrust is achieved when the incident beam is confined to the reflective part of the aperture, and it rapidly diminishes by increasing the spot size. We have done a comparative study to clarify further the role of substituting the reflective portions with transmissive ones, presented in the section S2 of the Supporting Information.

### Motion trajectory

In order to render the actual nonlinear system stable, one should conduct the motion trajectory study over the propulsion timescale to analyze the effects of relative ratios of displacement and rotation. Therefore, it is crucial to assess the mechanical performance of the system under initial displacement and rotation to estimate the acceleration, velocity, and amplitude of residual motion. As the sail accelerates, the major effect of the dispersion of the metasail building blocks, which yields fluctuating amplitude of displacement and rotation, can be unfolded by driving the in-plane forces ($F_x$) and torques ($\tau _y$) for different numbers of displacement ($\Delta x$) and rotation ($\Delta \theta$) offsets. The illumination beam is considered an unpolarized uni-modal Gaussian with a beam spot size of $w_0 = 0.8$ m and total power of $P_t\;=\;100\;\text {GW}$ to efficiently cover the aperture of the sail and minimize the energy spill-over. Here we are considering that a parabolic phase gradient with a focal distance of $F_0\;=\;0.93$ m is imprinted across both the reflective and transmissive metasurfaces with reflective metasurface radius of $d_p\;=\;0.8$ m. The payload, which is assumed to have a mass equal to the lightsail, is located $2d_{\text {CM}}\;=\;4\;\text {m}$ away from the sail in the illumination side where $d_{\text {CM}}\;=\;2\;\text {m}$. The total mass of the nanocraft is estimated as $m_t\;=\;5.32\;\text {g}$ where $m_T\;=\;m_{\text {sail}}\;+\;m_{\text {Starchip}}$.

**Figure 5:**
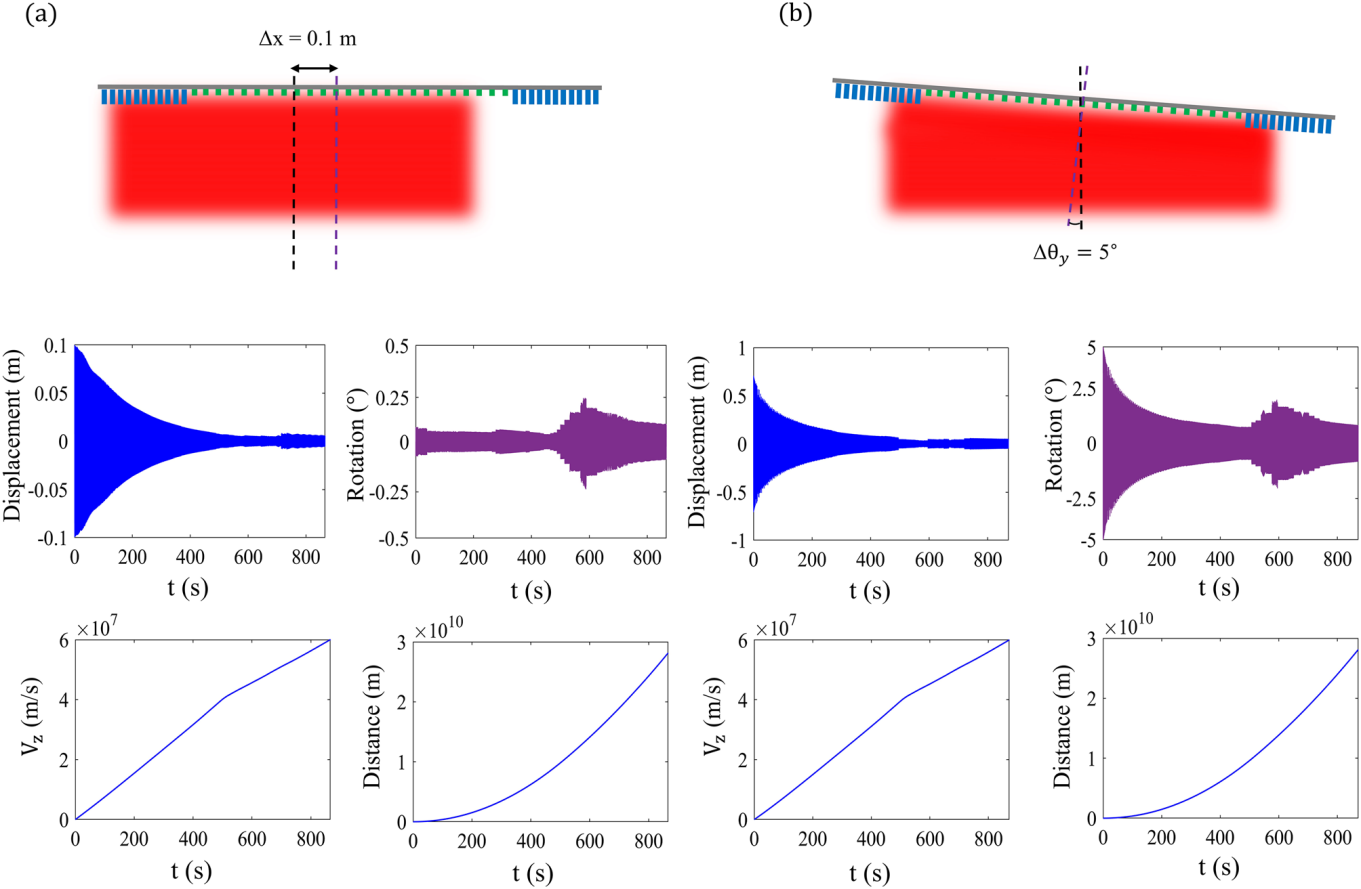
(**a**) The calculated displacement, rotation, velocity and travelled distance of a $2\times 2\;\text {m}^2$ dual-pattern parabolic metasail with $d_p\;=\;0.8$ m and $F_0\;=\;0.93$ m driven by 100 GW uni-modal laser beam, subject to initial displacement of $\Delta x\;=\;10\;\text {cm}$ with respect to the incident multi-modal beam. (**b**) depicts the same scenario for a dual-pattern sail with initial tilt of $\Delta \theta _y\;=\;5^\circ$ with respect to the upright position. Generated by MATLAB.

Figure 4h, i depict the $F_x$, and $\tau _y$ components of the in-plane imparted force to the lightsail in the Doppler-broadened propulsion band as a function of displacement offset along the $x-\text {axis}$ respectively. As it can be observed, the value of $F_x$ and $\tau _y$ possess a negative sign of the corresponding displacement offset, indicating the presence of strong restoring force and counter-balancing torque, respectively. It is also observed that there is a sudden surge in the values of $F_x$ and $\tau _y$ due to the hyper-geometric dispersion caused by the non-monotonic change in the phase response of the reflective unitcells. Figure 4j,k shows the generated restoring force and torque upon a rotation offset $\Delta \theta _y$ from the upright position. According to Fig. 4j,k, the generated force and torque counteract the tilts effectively, paving the way toward a self-stabilized beam-riding. It is envisioned that the value of the restoring forces and counter-balancing torques demonstrate linear dependency on the displacement and rotation offsets in the vicinity of the equilibrium position, which verifies the adopted linear stability analysis technique. The motion-trajectory analysis is done by forward integrating the Newton–Euler nonlinear equations in time wherein the local forces and torques are dependent on the velocity and the orientation of the lightsail. Figure 5a depicts the transverse displacement $\Delta x$, tilt $\Delta \theta _y$, velocity $v_z$, and traveled distance of the sail as a function of the propulsion time under an initial displacement of $\Delta _x\;=\;10\;\text {cm}$. As it can be observed, the amplitude of the lateral displacement of the sail has strongly diminished throughout the Doppler-broadened propulsion band as the value of the displacement has reached approximately near zero at the terminal velocity of $v_f\;=\;0.2c$. Nevertheless, the value of the rotation around the y-axis $\Delta \theta _y$ has not coupled to the displacement and has remained bounded to small values. Unlike the conventional metasails where the displacement and rotation were strongly coupled, here, the isolation between them is enabled by adding the transmissive metasurface at the outer region of the sail. It should be noted that the sudden growth in the amplitude of rotation is attributed to the jump in the phase response of the reflective metasurface discussed before. According to the figure, the sail reaches the target velocity of $v_f\;=\;0.2$ c in $t\;=\;832\;\text {s}$ while traveling $d_\text {acc}\;=\;28.6\;\text {Gm}$. Similarly, Fig. 5b illustrates the displacement, rotation, velocity $v_z$, and traveled distance of the metasail under initial rotation of the $\theta _y\;=\;5^\circ$. The result in Fig. 5b delineates the sail’s durability to large displacements along the $x-\text {axis}$ where the amplitude of lateral motion is strongly weakened thanks to the imparted restoring forces by the parabolic metasail. Furthermore, the generated counter-balancing torque has decreased the rotation amplitude to a relatively small value. It should be noted that the most dangerous scenario for the motion-trajectory study has a non-zero initial rotation where the coupling between the rotation and displacement can generate a strong imparted force at the edges of the sail leading to deviation of the sail from its path. In the current configuration, this coupling is minimized by replacing the reflective with transmissive elements. It can be observed that the metasail reaches the target velocity of $v_t\;=\;0.2$ c in 852 s. Engineering the coupling between the displacement and rotation plays a crucial role in realizing a self-stabilizing metasail that can successfully return to its equilibrium position, which is necessary for preventing persistent residual motion, which can lead to deviation of the sail from its path at the end of the propulsion time. The obtained results verify the effectiveness of our design in making the sail robust against the initial disturbing conditions. It has been shown earlier that by decreasing the radius of the reflective metasurface portion $d_p$, the marginal stability grows. A ray-tracing study for illustration of the role of parabolic phase gradient is conducted in the section S1 of the Supporting Information.

## Dual-pattern metasail with multi-modal beams

Illuminating a lightsail that has its payload at the back-side by a multi-modal Gaussian beam and engineering the phase gradient profile of the lightsail can mimic the scattering from a reflective sphere, enabling the self-stabilizing mechanism^2^. This kind of configuration brings numerous benefits, such as protection of the Starchip from the high-power laser beam, which can increase its temperature up to melting points and seriously damage the onboard equipment. In addition, as the illumination beam consists of four Gaussian beams, it can enable a time-varying dynamic propulsion beam to improve the stability criteria further. In this context, we have assumed that the beam is illuminated by a multi-modal Gaussian beam which consists of four Gaussian components given by:

$$\begin{aligned} E(x,y)= & {} \frac{2}{w^\prime _0}\sqrt{\frac{\eta P_1}{\pi }}\exp (\frac{-(x+\gamma )^2-(y+\gamma )^2}{w_0^{\prime 2}})+\frac{2}{w^\prime _0}\sqrt{\frac{\eta P_1}{\pi }}\exp (\frac{-(x-\gamma )^2-(y-\gamma )^2}{w_0^{\prime 2}})\\{} & {} +\frac{2}{w^\prime _0}\sqrt{\frac{\eta P_1}{\pi }}\exp (\frac{-(x+\gamma )^2-(y-\gamma )^2}{w_0^{\prime 2}})+\frac{2}{w^\prime _0}\sqrt{\frac{\eta P_1}{\pi }}\exp (\frac{-(x-\gamma )^2-(y+\gamma )^2}{w_0^{\prime 2}}) \end{aligned}$$

where the parameter $\gamma$ indicates the distance between their maximums and $P_1\;=\;(P_0)/4$ is the power that each Gaussian beam contains and $w^\prime _0$ is the beam spot size of the Gaussian components.

**Figure 6:**
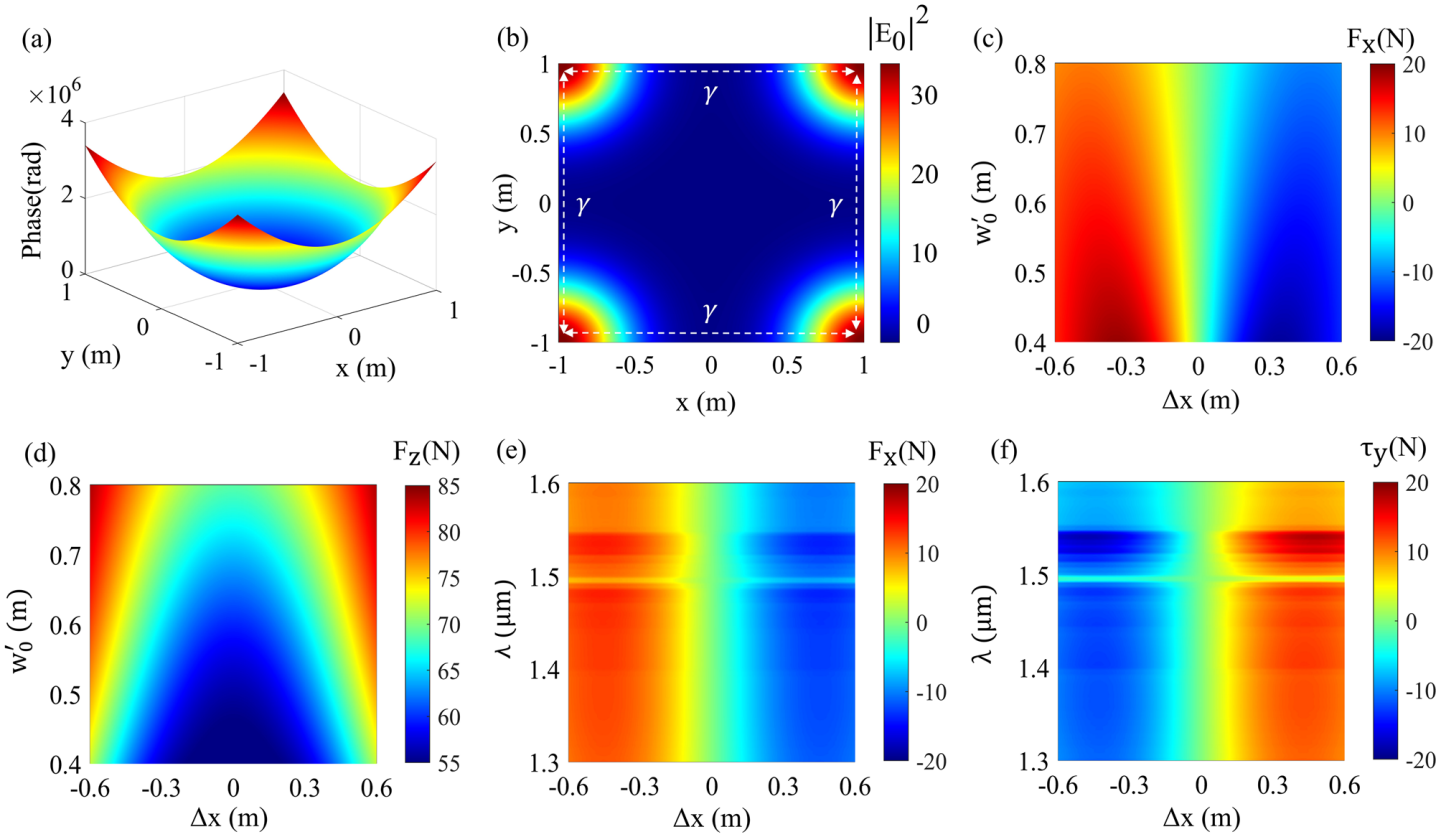
(**a**) The imprinted phase gradient on the metasail elements, where the inner side (reflective) and outer (transmissive) are diffractive spherical and parabolic respectively. (**b**) The intensity profile of the incident laser beam consisting of four Gaussian beams at the corners of the sail. (**c**,**d**) The imparted thrust and $F_x$ component of induced force as a function of beam spot size and the displacement. (**e**,**f**) Illustrate the $F_x$ and $\tau _y$ component of the induced optical force and torque as a function of the wavelength and the displacement, respectively. Generated by MATLAB.

Generally, when the center of mass is at the backside of the sail, a uni-modal Gaussian beam cannot stabilize the beam, and instead, it requires a multi-modal beam that has its maximums near the edges of the sail. Again, in this case, there are two sets of elements where in the central and outer portions, reflective and transmissive unitcells are placed, respectively. The maximums of the multi-modal beams are placed at the corners of the lightsail to enable self-stabilized beam-riding stability. The imprinted phase gradient on the reflective unitcells is a sphere with a radius of $R_s\;=\;1.43\;\text {m}$ given by $\varphi _s(x,y)=\frac{2\pi }{\lambda _0}(R_{\text {s}}-\sqrt{R^2_{\text {s}}-x^2-y^2)}$ where $\lambda _0\;=\;1.35\;\upmu \text {m}$. It should be noted that by defining the spherical phase with the mentioned radius, which is approximately equal to half of the diameter of square $D_{\text {square}}/2\;=\;1.43\;\text {m}$, the incident angle to some parts of the sail will be greater than the critical angle hence the beam will become evanescent, according to the Generalized Snell’s law. Consequently, the imprinted phase gradient will not operate in those portions. In order to prevent this phenomenon, we have substituted the reflective elements with transmissive ones in the region where $R_{\text {cs}}\;>\;0.95\;\text {m}$ where $R_{\text {cs}}$ is the distance from the center of the sail. More precisely, to minimize the discontinuity in the phase profile of the sail, which is detrimental as we have discussed previously, the added transmissive unitcells own a diffractive parabolic phase given by $\varphi (x,y)=+\frac{2\pi }{\lambda _0}(\sqrt{x^2+y^2+F_{\text {sp}}}-F_{\text {sp}})$ with a focal distance of $F_{\text {sp}}\;=\;1\;\text {m}$. Figure 6a,b show the imprinted phase gradient and the beam profile of the proposed configuration, respectively. As can be observed, the phase profile of the sail consists of two sections; the central portion has a spherical phase gradient, whereas the outer portion owns a parabolic phase gradient, as mentioned earlier. In this scenario, if the metasail is displaced with respect to the incident laser beam, the transmissive and reflective metasurfaces will generate a restoring force in the opposite direction, pushing the sail to the beam’s central axis, as depicted in Fig. 6c. According to the figure, the $F_x$ component of the induced force is in the opposite direction of the displacement, which is a requirement for a stable beam-riding. By decreasing the beam spot size, the induced lateral force increases as, in this case, the maximums of the incident Gaussian components of the beam will be more concentrated in the transmissive metasurfaces, which generate larger in-plane forces due to their diffractive parabolic phase gradient. However, decreasing the spot size $w^\prime _0$ of the propulsion beam decreases the net thrust as depicted in Fig. 6d, which imposes a trade-off between the acceleration and the durability of the sail against the in-plane displacements. In order to clarify the self-stabilizing behavior of the sail, we have plotted the $F_x$ component of the imparted force and $\tau _y$ component of the generated torque as a function of displacement for the wavelengths within the propulsion band, as shown in Fig. 6e,f, respectively. Both figures clearly show the generation of the restoring force and counterbalancing torque for the positive and negative displacement of the sail with respect to the multi-modal propulsion beam. Also, as it can be observed, the lightsail preserves its broadband functionality throughout the Doppler-broadened propulsion spectrum as the variation in the amplitude of generated components of the force and torque is relatively small for specific displacement except for a narrow portion of the band which is due to the abrupt phase change of reflective elements in the GMR branch. It should be noted that the upright position is rendered by the position where the center of the sail coincides with the center of the multi-modal beam profile, as depicted in the figures where the induced lateral force and torque are zero. A beam-tracing study is conducted in the section S1 of the Supporting Information to better illustrate the functionality of each phase gradient in two different wavelengths in the Doppler-broadened propulsion band.

**Figure 7:**
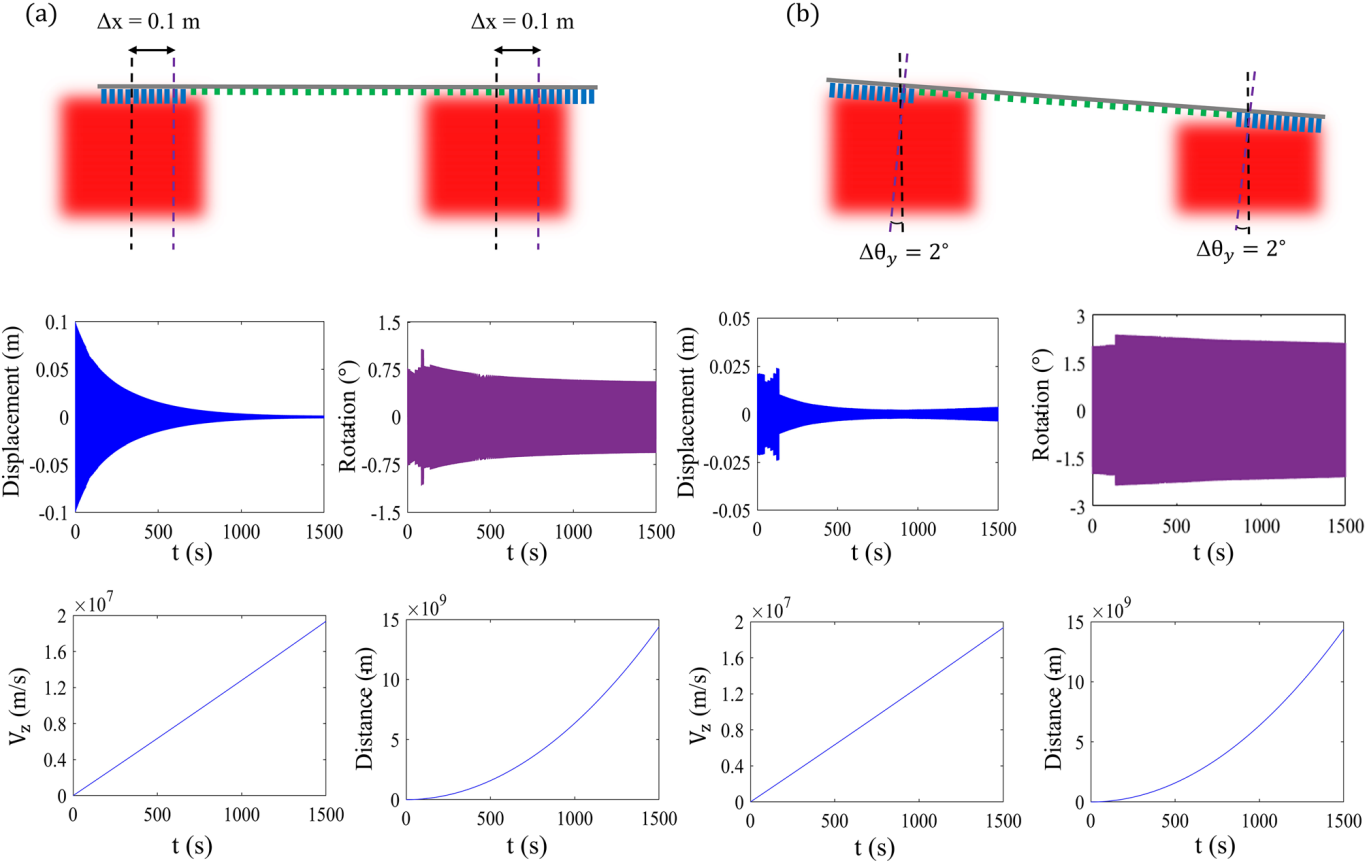
(**a**) The calculated displacement, rotation, velocity and travelled distance of a $2\times 2\;\text {m}^2$ dual-pattern spherical-parabolic metasail subject to initial displacement of $\Delta x\;=\;10\;\text {cm}$ with respect to the incident multi-modal beam. (**b**) depicts the same scenario for a dual-pattern sail with initial tilt of $\Delta \theta _y\;=\;2^\circ$ with respect to the upright position. Generated by MATLAB.

### Motion trajectory

Similar to the case where the incident beam was a uni-modal Gaussian, by considering the nanocraft as a rigid body, the motion trajectory of the sail in the global coordinate system can be estimated by solving the nonlinear Newton–Euler’s system of equations for a small displacement and angular rotations. Here we have assumed the sail’s dimensions as 2$\times$2 $\text {m}^2$ with a total mass of $\text {m}_\text {T}\;=\;4.7\;\text {g}$ and $\text {d}_\text {p}\;=\;0.95\;\text {m}$ where the metasail and the payload have an equal mass. The incident laser beam is assumed to have a multi-modal Gaussian intensity profile with $\gamma \;=\;1\;\text {m}$ and a total net power of 100 GW divided by four equal power Gaussian components with $w^\prime _0\;=\;0.6\;\text {m}$. The center of mass is located at $d_{\text {CM}}\;=\;3\;\text {m}$ in the back-side of the sail for protecting the Starchip from the high-power propulsion beam. It should be noted that during this analysis, we have assumed that the distance between the four Gaussian components of the propulsion laser beam is unchanged, thanks to the utilization of adaptive optics. In order to manifest the real behavior of the sail motion trajectory, we have conducted the simulation for two different cases; wherein the first scenario, the sail has an initial displacement with respect to the center of the multi-modal beam axis, and in the second case the sail has initial elevation with respect to the upright position. Figure 7a depicts the calculated displacement, rotation, velocity, and traveled distance for the case where the sail has an initial displacement offset of $\Delta x\;=\;0.1\;\text {m}$ with zero elevation. As can be observed, the oscillatory transverse motion of the sail remains bounded throughout the acceleration period while its amplitude diminishes by moving forward in the time frame, reaching zero at the end of the acceleration. Due to the coupling between displacement and rotation, the initial displacement of the sail induces a rotational motion that remains bounded during the entire acceleration period. Nevertheless, the velocity of the spacecraft reaches $v_z\;\approx \;2\times 10^7\;\text {ms}^{-1}$ in $t\;=\;1500\;\text {s}$ of acceleration which corresponds to the Doppler-broadened wavelength of $\lambda \;=\;1.39\;\upmu \text {m}$. Higher speeds up to the terminal velocity of $v_t\;=\;0.2$ c can be attained by continuing the motion trajectory, although the required distance for acceleration may require a larger terrestrial laser array for keeping the beam diffraction-limited. It should be remarked that the significant reduction in the acceleration performance is mainly due to adjusting the incident beam to the edges of the sail, as a quarter of the total beam power is incident on the sail’s aperture. Using multi-modal beams as a propellant, however, imposes an important trade-off between the launch energy and acceleration time of the sail. Figure 7b illustrates the estimated displacement, rotation, velocity, and traveled distance for the case where the sail has an initial tilt of $\Delta \theta \;=\;2^\circ$ with respect to the upright position. As can be observed in this case, the coupling between the rotation and displacement is not significant as the amplitude of the in-plane displacement remains bounded, shrinking to very small values at the end of the acceleration period. Nevertheless, the amplitude of the sail rotation remains bounded throughout the propulsion period. Furthermore, the obtained velocity and traveled distance results are similar to the previous case, indicating the negligible effect of the initial position and orientation of the sail on the final speed and traveled distance of the sail. It should be noted that similar results can be obtained for the displacements along the y-axis and rotation around the x-axis, considering the symmetry of the problem. More information on the tolerance of the sail against the presence of both initial displacement and rotation is brought in section S1 of the supporting material.

As a final remark, we have conveyed optomechanical study for two novel configurations of nanocraft, and it is envisioned that the achieved results for dynamics of the sail can pave the way toward the realization of photon-assisted self-stabilized lightsails; however, significant challenges regarding the fabrication and thermal management of the sail is expected to be investigated. Although it has been shown that a silica layer with sufficient thickness can keep the temperature of the sail below the melting points due to its high emissivity in the near-infrared region, engineering the thermal emissivity of the nanoscale patterns is also investigated^71–73^. Nevertheless, the non-rigidity of the real lightsail structure and its effects on the motion trajectory of the sail can properly be modeled by Lagrangian mechanics^74,75^. The non-rigidity of the all-dielectric platform will result in the emergence of local deformations of the thin structure, which can be taken into account as one of the optimization parameters in conformal metasurfaces^36,76,77^.

## Conclusion

In this work, we have considered a dual-pattern metasail consisting of reflective and transmissive building blocks in an all-dielectric Si-silica platform. It is demonstrated that surrounding the parabolic reflective metasurface with parabolic transmissive elements can increase the marginal stability of the relativistic lightsail driven by the radiation pressure of a high-power laser beam. Added transmissive portions generate counterbalancing torque instead of pushing the sail away in the case of displacement of the metasail with respect to the propulsion beam, which increases the stability degree of the sail. A multi-objective genetic algorithm (GA) optimization is applied to obtain a near-optimal trade-off between the stability degree and net thrust of the lightsail. The optimized structure is not only able to effectively damp the oscillatory motion but also can decrease the required minimum center of mass distance, which is of great importance toward the realization of gram-scale nanocrafts. Next, we have proposed a novel configuration of the spacecraft where the central and outer building blocks have reflective and transmissive phase gradients, respectively, in which the payload is placed at the back-side of the lightsail to protect it from the high-power propulsion laser beam, which can severely damage the Starchip. In this case, a simple uni-modal Gaussian beam is not able to enable beam-riding stability. Instead, a multi-modal Gaussian beam, with its maximums at the corners of the sail, accelerates the lightsail. In this case, the calculated imparted forces and torques followed by a motion trajectory study verify the broadband functionality and its effect on extending the stability degree of freedom of nanocrafts.

## Supplementary Information

Supplementary Information.

## Data Availability

The datasets used and/or analysed during the current study available from the corresponding author on reasonable request.
