# Supplementary material for: Increasing the stability margins using multi-pattern metasails and multi-modal laser beams
Source: Sci Rep. 2022 Nov 21;12:20034. doi: 10.1038/s41598-022-24681-w (PMC9681836; doi:10.1038/s41598-022-24681-w)
Supplement: Supplementary file 1 — Supplementary Information. [file 41598_2022_24681_MOESM1_ESM.pdf]

Supporting Information: Increasing the Stability Margins using Multi-Pattern Metasails and Multi-Modal Laser Beams

Mohammadrasoul Taghavi[1] and Hossein Mosallaei[1]

[1]*Metamaterials Laboratory, Electrical and Computer Engineering Department, Northeastern University, Boston, MA 02115, USA,*

The supporting file is organized in 6 sections. In section 1 we further discuss the optomechanical modeling framework. In section 2, we perform a comparative stability analysis study for the proposed structure. In the section 3 we illustrate the full-wave simulation of designed unitcells for verifying the broadband functionality of the designed unitcells and also viability of the used approach for calculating optical forces. We also study the effect of fabrication errors in the performance of the beam-steering ability of the structure. In section 4 we discuss the angular sensitivity of the proposed dielectric unitcells. In section 5 further information on the tolerance of the metasail against the initial displacement and rotation is provided. In section 6 the effect of uncertainties in values of the loss in the performance of the sail is discussed.

S1. Further Information on Optomechanical Modeling

Here, we point out more information about the optomechanical method that is adopted for obtaining the exerted optical force to the large-scale metasail. All the results are generated using MATLAB[1]. Due to large size of the sail, the local imparted optical force to the group of the unitcells can be obtained by evaluating the flux of Maxwell's stress tensor through a box enclosing the group of elements. Moreover, the time averaged local optical force is given by:

$$\langle \overrightarrow{dF} \rangle = \frac{1}{2} Re\{\hat{n}_i.\overline{\overline{T}}_{\rm em} + \hat{n}_t.\overline{\overline{T}}_{\rm em}\} dS \tag{1}$$

Where $\hat{n}_t$ and $\hat{n}_i$ are the normal vectors for the transmission and illumination side surfaces, respectively. $\overline{\overline{T}}_{\rm em}$ is Maxwell's stress tensor which can be written as:

$$\overline{\overline{T}}_{\rm em} = \overrightarrow{D}\overrightarrow{E} + \overrightarrow{B}\overrightarrow{H} - \frac{1}{2}\overline{\overline{I}}_{3\times3}(\overrightarrow{D}.\overrightarrow{E} + \overrightarrow{B}.\overrightarrow{H}) \tag{2}$$

in which the $\overline{\overline{I}}_{3\times3}$ is a unity dyad and $\overrightarrow{H}$, $\overrightarrow{B}$, $\overrightarrow{D}$,$\overrightarrow{E}$ are the magnetic induction and field vectors, electric displacement and induction, respectively. As mentioned in manuscript, due to the unfeasibility of the full-wave simulation of the large-scale metasurface, by using the local periodicity assumption and expressing the total electric field as incident, reflected and transmitted components, the local optical force can be written as: $\langle \overrightarrow{dF} \rangle = \langle \overrightarrow{dF_i} \rangle + \langle \overrightarrow{dF_r} \rangle + \langle \overrightarrow{dF_t} \rangle$ which can be obtained as below:

$$\begin{aligned} \langle \overrightarrow{dF_i} \rangle = & \frac{\hat{z}}{2}\epsilon_0 |E_i(x,y)|^2 \cos^2(\theta_i) dS \\ & + \frac{\hat{x}}{2}\epsilon_0 |E_i(x,y)|^2 \cos\theta_i \sin\theta_i \cos\varphi_i dS \\ & + \frac{\hat{y}}{2}\epsilon_0 |E_i(x,y)|^2 \cos\theta_i \sin\theta_i \sin\varphi_i dS \end{aligned} \tag{3}$$

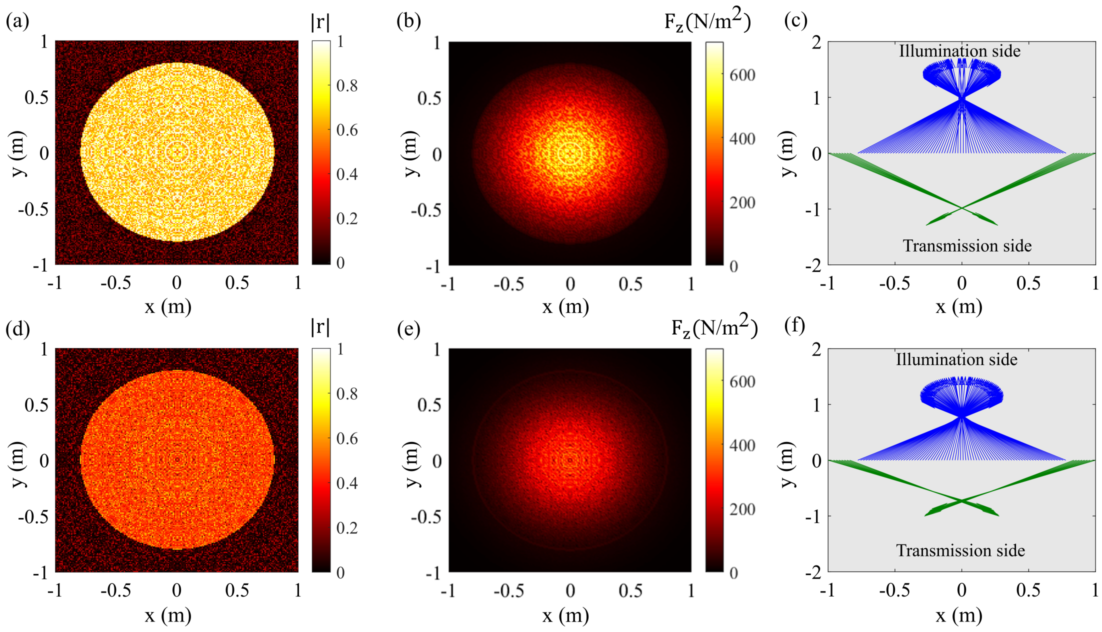

Supplementary Figure S 1: The spatial distribution of (a,d) reflection amplitude, (b,e) $z-$component of the imparted force, (c,f) beam-tracing results for dual-pattern metasail under illumination of a uni-modal un-polarized laser beam at $\lambda = 1.35$ μm) and $\lambda = 1.55$ μm) , respectively.

$$\begin{aligned}\langle\overrightarrow{dF_r}\rangle &= \frac{\hat{z}}{2}\epsilon_0|E_i(x,y)|^2|r(x,y,\lambda,\theta_i,\varphi_i)|^2\cos^2(\theta_r)dS \\ &-\frac{\hat{x}}{2}\epsilon_0|E_i(x,y)|^2|r(x,y,\lambda,\theta_i,\varphi_i)|^2\cos(\theta_r)\sin(\theta_r)\cos(\varphi_r)dS \\ &-\frac{\hat{y}}{2}\epsilon_0|E_i(x,y)|^2|r(x,y,\lambda,\theta_i,\varphi_i)|^2\cos(\theta_r)\sin(\theta_r)\sin(\varphi_r)dS\end{aligned} \tag{4}$$

$$\begin{aligned}\langle\overrightarrow{dF_t}\rangle &= -\frac{\hat{z}}{2}\epsilon_0|E_i(x,y)|^2|t(x,y,\lambda,\theta_i,\varphi_i)|^2\cos^2(\theta_t)dS \\ &-\frac{\hat{x}}{2}\epsilon_0|E_i(x,y)|^2|t(x,y,\lambda,\theta_i,\varphi_i)|^2\cos(\theta_t)\sin(\theta_t)\cos(\varphi_t)dS \\ &-\frac{\hat{y}}{2}\epsilon_0|E_i(x,y)|^2|t(x,y,\lambda,\theta_i,\varphi_i)|^2\cos(\theta_t)\sin(\theta_t)\sin(\varphi_t)dS\end{aligned} \tag{5}$$

where $|r|$ and $|t|$ are the local reflection and transmission amplitudes and $|E_i|$ is the amplitude of the local incident electric field and θ and ϕ are the polar and azimuth angles, respectively. Next, the generalized Snell's law can be used to calculate the anomalous reflection and transmission angles as:

$$\sin(\theta_{r,t})\cos(\varphi_{r,t}) = -\frac{1}{k_0}\frac{\partial\Phi_{r,t}(x,y,\lambda,\theta_i,\varphi_i)}{\partial_x} + \sin(\theta_i)\cos(\varphi_i) \tag{6}$$

$$\sin(\theta_{r,t})\sin(\varphi_{r,t}) = -\frac{1}{k_0}\frac{\partial\Phi_{r,t}(x,y,\lambda,\theta_i,\varphi_i)}{\partial_y} + \sin(\theta_i)\sin(\varphi_i) \tag{7}$$

Where φ is the spatially-variant phase imprinted across the metasurface. Using the above formulation one can calculate the exerted optical force on the large-scale metasurface with a good approximation. Supplementary Figure S1(a,d) illustrate the spatial distribution of the reflection amplitude across the parabolic dual-pattern metasail at two different wavelengths of $\lambda = 1.35$ μm and $\lambda = 1.55$ μm throughout the Doppler-broadened propulsion spectrum. As it can be observed from the results for $\lambda = 1.35$ μm, the central section where reflective elements are present, the reflection amplitude remains high whereas the transmissive layers at the edges own a very low reflection amplitude which is essential to prevent coupling between the displacement and rotation. According to the results at $\lambda = 1.55$ μm the reflection amplitude has decreased for the central reflection portion as

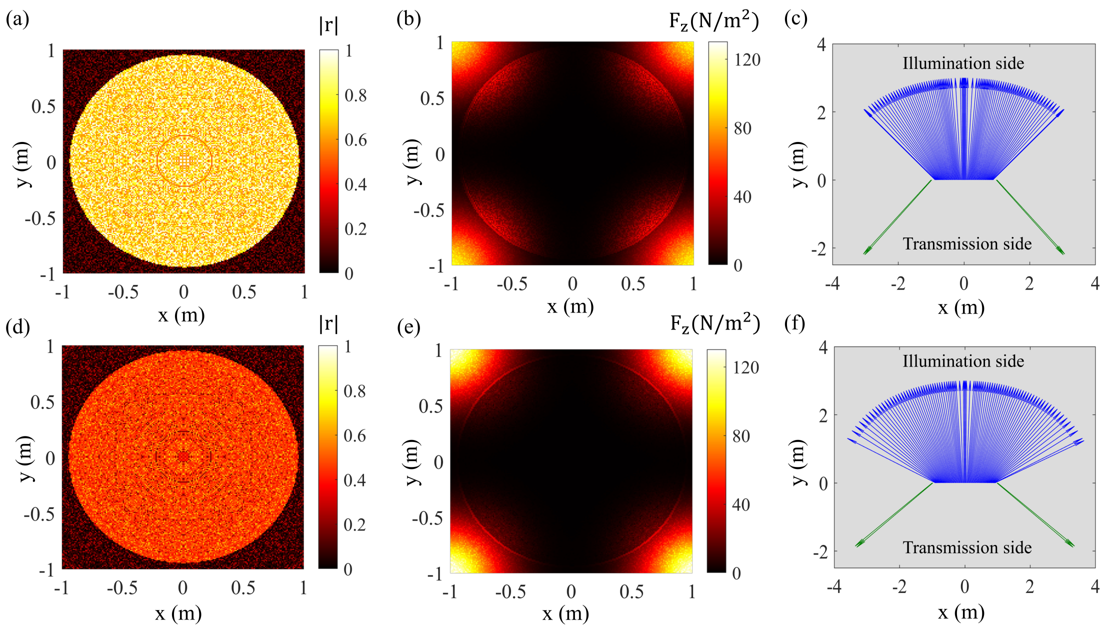

Supplementary Figure S 2: The spatial distribution of (a,d) reflection amplitude, (b,e) $z-$component of the imparted force, (c,f) beam-tracing results for dual-pattern metasail under illumination of a multi-modal unpolarized laser beam at $\lambda = 1.35$ μm and $\lambda = 1.55$ μm) , respectively.

a result of presence of non-perfect response of the reflective metasurface at this wavelength. However, the reflection amplitude for the transmissive portion remains at very low values thanks to the perfect broadband characteristics of the tall transmissive elements. Supplementary Figure S1(b,e) depict the spatial distribution of the z-component of the exerted force, multiplied to the $L^2/(Ns)^2$ where $L = 2$ m and $Ns = 300$ are the dimension and the number of chosen samples over the sail, respectively, to the sail for two different wavelengths of $\lambda = 1.35$ μm and $\lambda = 1.55$ μm. Here, the illumination beam is a simple Gaussian with the beam spot size of $w_0 = 0.8$ and consequently the response of the reflective metasurface mainly determines the amplitude of the F_z component of the exerted force. It is clear from the figures that there is a significant reduction in the amplitude of the imparted force as a result of reduction of the reflection amplitude of the reflective metasurface at the wavelengths near to the end of the Doppler-broadened propulsion spectrum. In order to gain a better understanding of the functionality of the sail, the direction of the reflected and transmitted beams for the wavelengths of $\lambda = 1.35$ μm and $\lambda = 1.55$ μm are calculated via ray-tracing based on the generalized Snell's law and depicted at the Supplementary Figure (c,f). The obtained results, clearly indicate that despite the induced chromatic aberrations and limited phase coverage of the reflective unitcells which has led to the reduction of focal length at higher wavelength, the direction of the reflected beams points toward the central axis of the metasail which is necessary for generation of in-plane restoring forces and counter-balancing torques. The similar results are obtained at Supplementary Figure S2 for the case where the Starchip is placed at the back-side of the sail with the illumination beam being a multi-modal Gaussian. As discussed in the manuscript, in this case the reflective metasurface has a spherical phase profile with radius of $R = 1.4$ m and the transmissive portion at the edges has a diffractive parabolic phase profile with focal distance of $F_t = 1$ m. According to the Supplementary Figure S2(a,d) the amplitude of the spatially distributed reflection coefficient has decreased as a result of non-ideal response of reflective metasurface at higher wavelength. Supplementary Figure S2(b,e) depict the exerted force along the $z-$axis, multiplied to the $L^2/(Ns)^2$ where $L = 2$ m and $Ns = 300$ are the dimension and the number of chosen samples over the sail, respectively for the dual-pattern sail under illumination of a unpolarized multi-modal Gaussian beam with $\gamma = 1$ m. The amplitude of induced force has a large value for the corner sections as the maximum of the beam is place there. Although the transmissive elements have a low reflection amplitude, the amplitude of the beam is high enough to exert acceptable amount of force to drive the sail. Supplementary Figure S2(c,f) depict the beam-tracing results for this case which

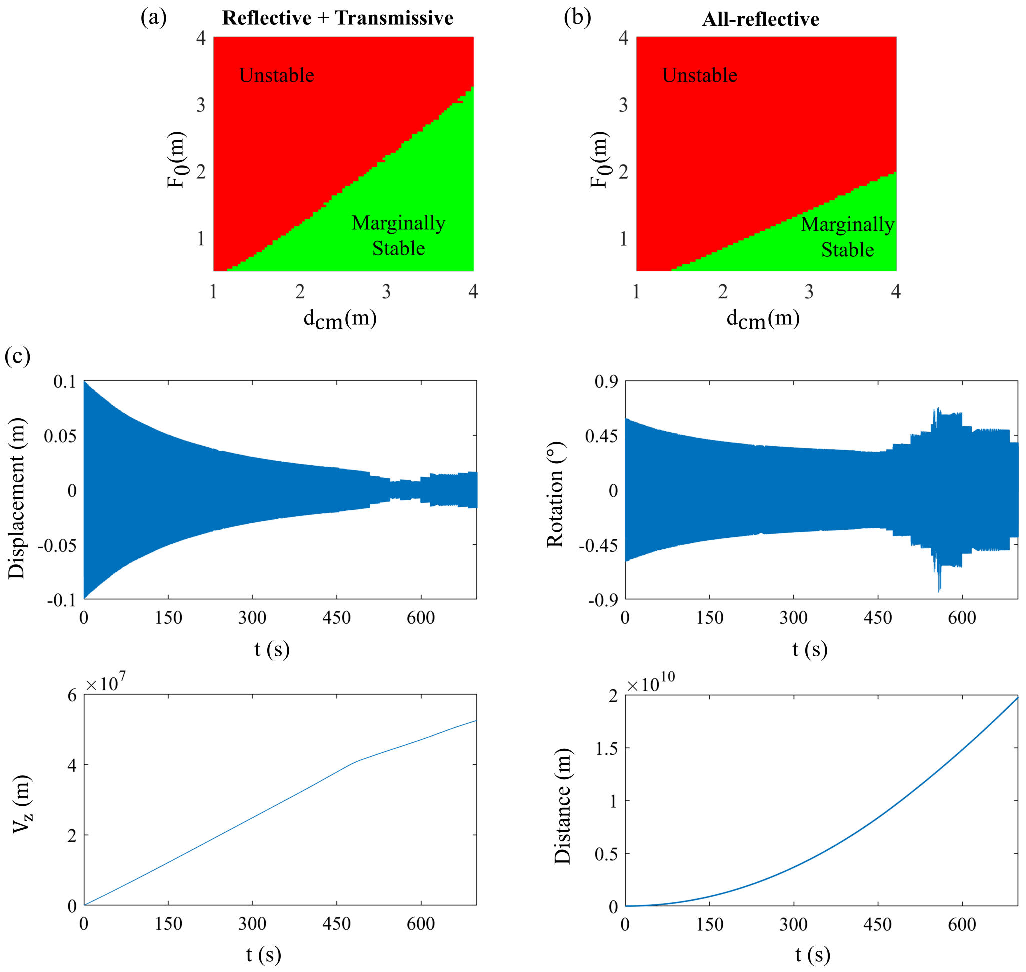

Supplementary Figure S 3: (a,b) The marginal stability condition as a function of focal distance of the imprinted phase gradient and the center of mass distance of the spacecraft with $d_p = 0.8$ m and $d_p = 0$ m, respectively. (c) The calculated displacement, rotation, velocity and travelled distance of a all-reflective sail with $d_{\mathrm{CM}} = 4$ m subject to initial displacement of $\Delta x = 10$ cm.

clearly show the direction of the reflected beams from the diffractive spherical metasail at the $\lambda = 1.35$ μm and $\lambda = 1.55$ μm. The chromatic dispersion due to the red-shift of the propulsion wavelength leads to the angular expansion of the reflected beams. Nevertheless, the transmitted component of the fields from the edge transmissive elements are shown at the same wavelengths, experiencing a chromatic dispersion.

S2. Comparative Study

Here, we have done a comparative study to further demonstrate the effects of substituting reflective portions by transmissive metasurfaces on the stability analysis of the metasail. As discussed in the manuscript, minimizing the cross-coupling between the displacement and rotation is the most important parameter in the stability analysis which can be achieved by adding a transmissive layer to the corners of the structure followed by a proper multi-objective optimization. Furthermore, any initial displacement and rotation will lead to abruptly imparted torque, deviating the sail away from its path. In order to directly visualize the effect of addition of transmissive layer to the stability of the nanocraft, we have plotted the marginal stability conditions for a dual-pattern sail with $d_p = 0.8$ m and all-reflective sail in Supplementary Figure S3(a,b), respectively. As it can be observed, the marginal stability of the sail has been improved by substituting the reflective portions at the edges with transmissive patterns. This improvement stems from minimization of cross-coupling between the displacement and rotation as elements at the outer portions generate larger torque and using transmissive unitcells in those areas lead to generation of larger cross-coupling torque, helping the self-stabilizing mechanism.

Supplementary Figure S3(c) depicts the motion trajectory study of a sail which consists of only reflective unitcells with $d_{\text{CM}} = 4$ m. It should be noted that all-reflective metasail with $d_{\text{CM}} = 2$ m is not marginally stable hence we have chosen $d_{\text{CM}} = 4$ m for comparing the motion trajectory performance of a multi-pattern with an all-reflective single pattern metasail. As can be observed in the absence of transmissive elements, the amplitude of the oscillatory motion of the sail subject to initial displacement of $\Delta x = 10$ cm has increased as a result coupling between the displacement and rotation. Nevertheless, the velocity and travelled distance of the nanocraft has not significantly changed as large portion of incident propulsion beam stays mainly within the reflective pattern.

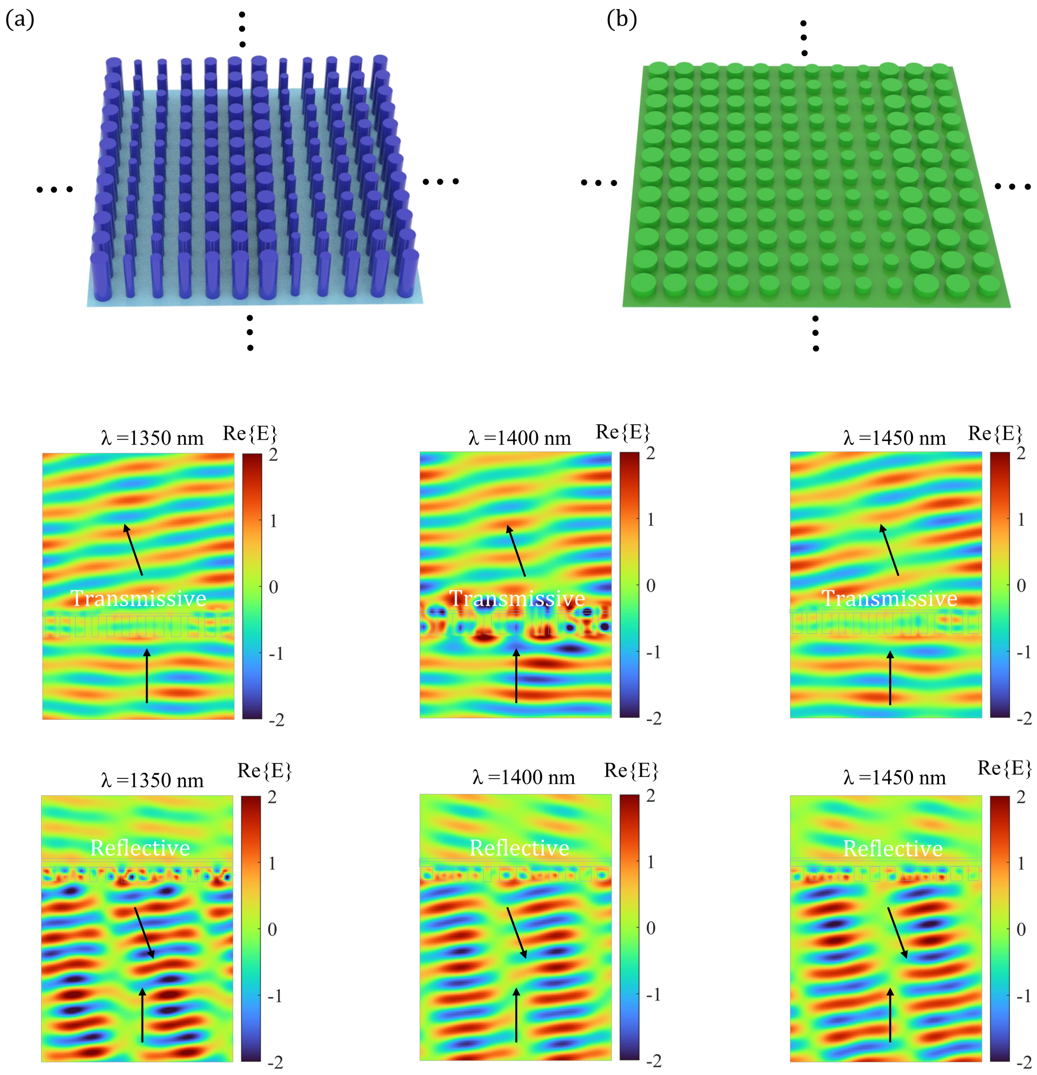

Supplementary Figure S 4: Depiction of supercells cosisting of twelve (a) Transmissive and (b) reflective all-dielectric unitcells and their full-wave RCWA simulation results, steering the transmitted and reflected wavefronts to $\theta_t = 20°$ and $\theta_r = 20°$ at $\lambda = 1.35$ μm, $\lambda = 1.4$ μm ,$\lambda = 1.45$ μm, respectively.

S3. Full-wave Simulations and Fabrication Tolerance

As discussed in the manuscript, due to the extensive computational complexity caused by the large size of the metasail, the optomechanical response of such large structure is obtained by adopting the generalized Snell's law under the assumption of local periodicity. Furthermore, in order to verify the broad-band functionality of the designed reflective and transmissive unitcells, we have done a full-wave rigorous coupled wave analysis (RCWA) simulations by considering a periodic arrangement of reflective and transmissive supercells each consisting of 12 unitcells along the $x-$axis at three chosen wavelengths, $\lambda = 1.35$ μm, $\lambda = 1.4$ μm ,$\lambda = 1.45$ μm ,across the Doppler-broadened propulsion spectrum. The imprinted phase over the reflective and transmissive supercell is set such that the reflected and transmitted beams are steered toward anomalous angle of $\theta_r = 20°$ and $\theta_t = 20°$.

Supplemntary Figure S4 depicts the obtained full-wave simulation for the reflective and transmissive supercells at three different wavelengths, respectively. As it can be observed from the results, the angle of steered wavefront($\theta_r = 20°$, $\theta_t = 20°$) is in a great agreement with the prediction of generalized Snell's law, indicating the accuracy of adopted method.

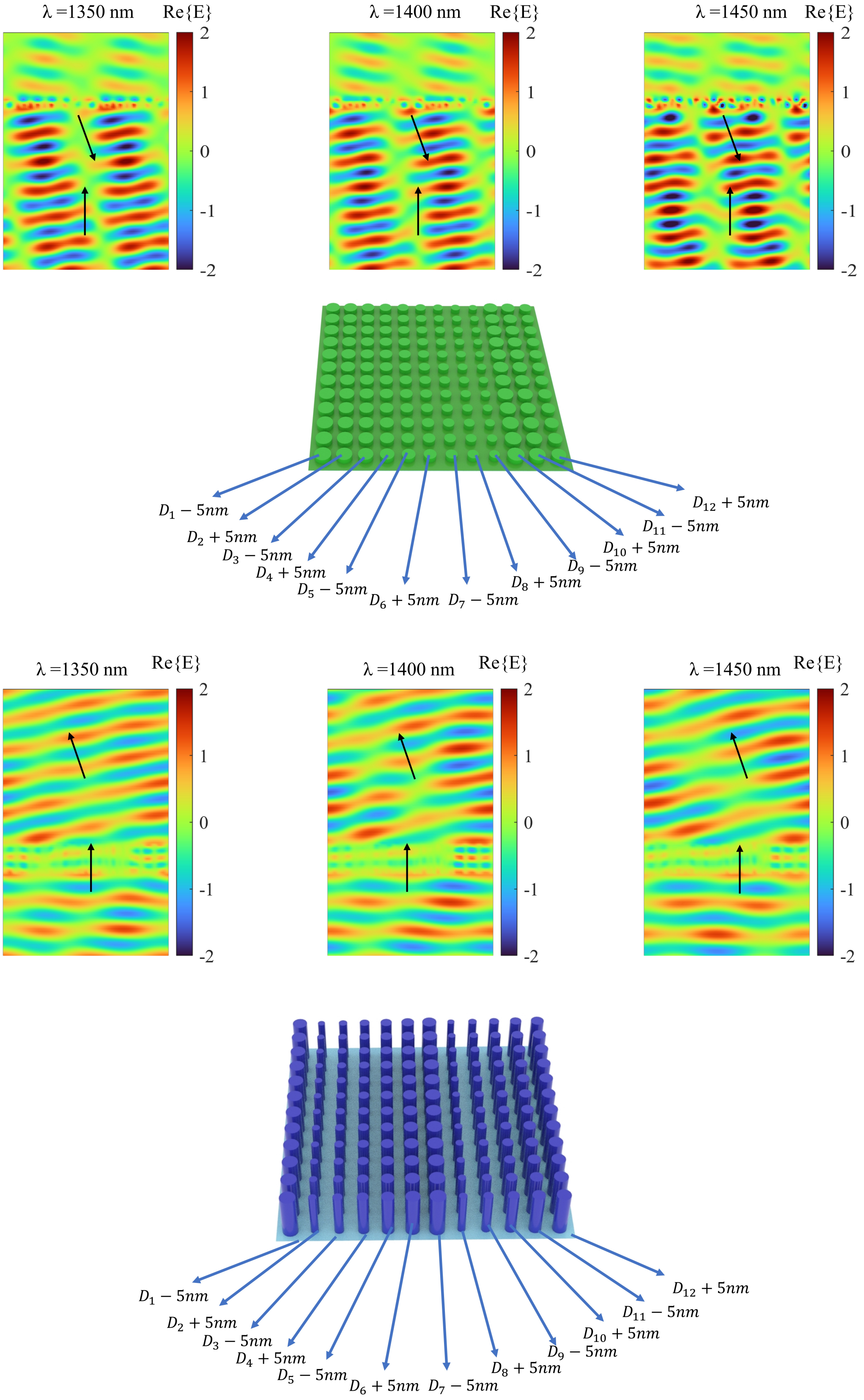

Supplementary Figure S 5: Depiction of modified supercells cosisting of twelve (a) Transmissive and (b) reflective all-dielectric unitcells and their full-wave RCWA simulation results, steering the transmitted and reflected wavefronts to $\theta_t = 20°$ and $\theta_r = 20°$ at $\lambda = 1.35\ \mu$m, $\lambda = 1.4\ \mu$m ,$\lambda = 1.45\ \mu$m, respectively. In this case, the $\Delta D = \pm 5$ nm variation of diameter of disks in the array is illustrated by arrows. It should be noted that the $D_{1\ldots12}$ are the diameters of the originally designed supercell in the previous part.

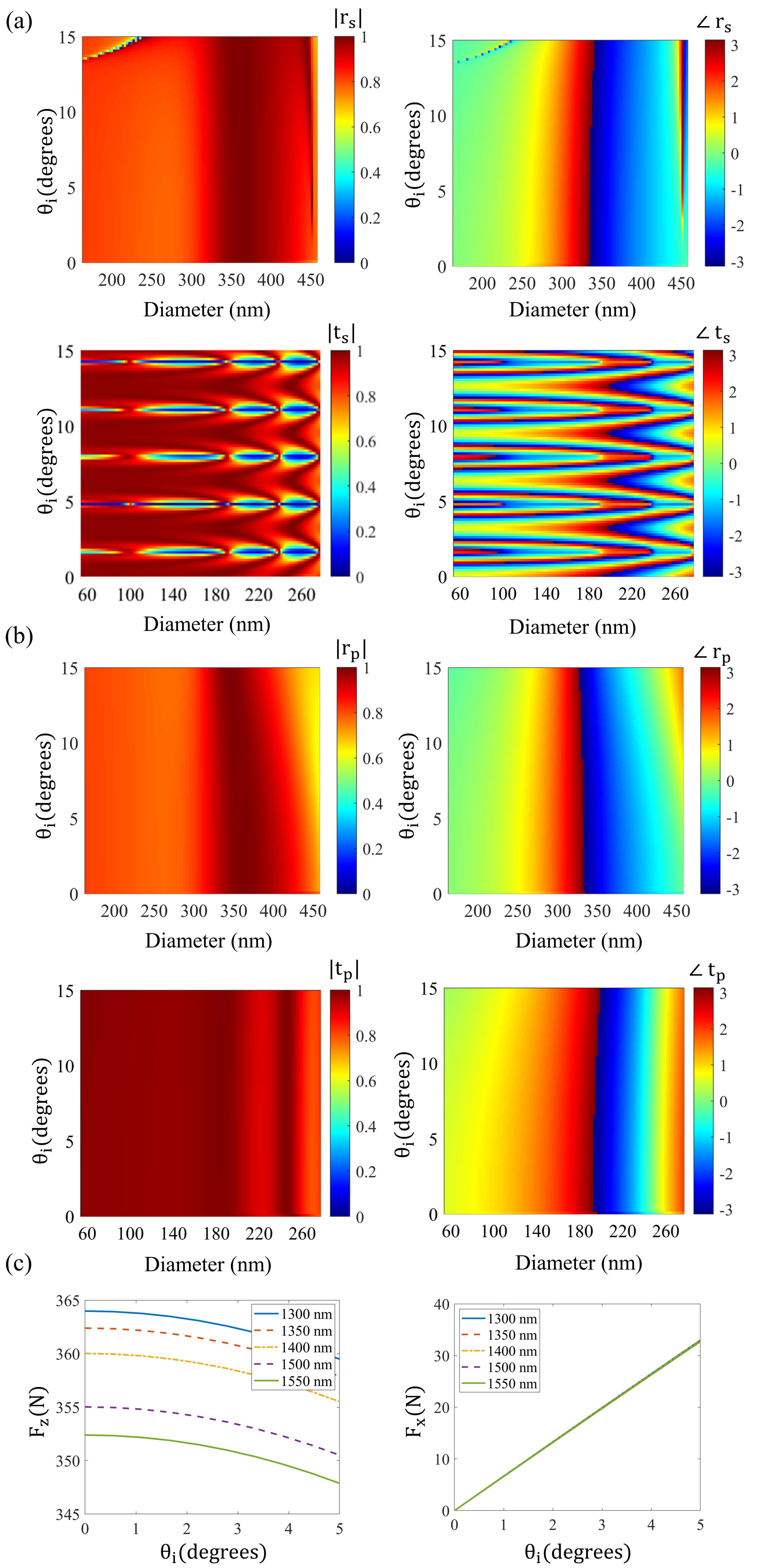

Supplementary Figure S 6: Calculated amplitude and phase of the reflection and transmission coefficients of reflective and transmissive unitcells for -s (a) and -p (b) polarized plane-wave using RCWA. (c) depicts the imparted F_x and F_z for deviation angles of $\theta_i = (0-5°)$.

However, the non-perfections observed in the steered wavefront are caused by the violation of the local-periodicity assumption as the desired phase is achieved by different diameters of the nanodisks for both of the reflective and transmissive supercells.

It should be remarked that although the local periodicity assumption near to the edge sections of the sail and in the boundary of the reflective and transmissive portions may not be satisfies well, the spurious scattering effect caused by them is expected to be negligible with respect to the response of the large structure of sail.

Next, in order to study effect of the imperfections in the metasail fabrication, we have altered the diameters of the previous reflective and transmissive arrays in a way that the elements experience a variation of ±5 nm in their originally designed diameters as shown in Supplementary Figure S5. This kind of variation in the amplitude of the diameter disrupts the phase profile of the array, required for perfect beam steering.

As can be seen from Supplementary Figure S5, both reflective and transmissive arrays, maintain their functionality even with presence of such large imperfection in their phase distribution. It should be noted that, the observed tolerance of the proposed all-dielectric unitcells stem from the relatively low quality factor of the exploited resonances.

S4. Angular Sensitivity of the Elements

Angular performance of the lightsail in critical orientations during the acceleration period highly depends on the sensitivity of the angular response of its nanophotonic elements. As mentioned in the main manuscript we have limited our study to maximum $\theta_y = 5°$ to keep the assumption of $\omega_{x,y,z} \approx \frac{d\theta_{x,y,z}dt}{}$ valid. However, for better illustrating the sensitivity of response of the elements to different oblique incidence angles we have plotted the amplitude and phase of the reflection/transmission coefficient of reflective/transmissive unitcells in Supplementary Figure S6(a,b). As can be seen, by increasing the oblique incident angle, reflective and transmissive unitcells maintain the high reflectivity and transmissivity. Large phase coverage for both unitcells is conserved even for modest deviation angles which renders the proposed elements ideal choices for such a mission. It should be noted that narrow reflection branches in the absolute value of the transmission coefficient of transmissive unitcells are observed as a result operation in hybrid Mie-Fabry-Perot. As observed from the Supplementary Figure S6, phase of the transmission coefficient of the same unitcell experiences variations. In our optomechanical analysis which is limited to maximum deviation angle of $\theta_y = 5°$, effect of such phase distortion is assumed to be negligible. Supplementary Figure S6(c) shows the calculated F_z and F_x considering the angular response of the unitcells for angles $\theta_i = (0 - 5°)$. As can be seen by increasing the deviation angle the imparted F_x increases monotonically for all of the selected wavelengths, indicating the negligible impact of angular dispersion of dielectric unitcells. Also, the computed F_z decreases with increasing the rotation angle. Nevertheless, by increasing the wavelength, the imparted net thrust F_z decreases as the reflectivity of the central reflective elements is reduced in higher wavelengths. According to the calculated forces, it is clear that for deviation angles considering in our analysis, the angular sensitivity of the unitcells can be safely neglected.

(a)

Dual-pattern Uni-modal Beam

$\Delta\theta_{max}$ (∘)	5	5	5	5	5	5	4.5	4	4	3.5	3	2.5	2	0
**Δx(m)**	**0**	**0.1**	**0.2**	**0.3**	**0.4**	**0.5**	**0.6**	**0.7**	**0.8**	**0.9**	**1.0**	**1.1**	**1.2**	**1.3**

(b)

Dual-pattern Multi-modal Beam

$\Delta\theta_{max}$ (∘)	5	5	4	3	3
**Δx(m)**	**0**	**0.1**	**0.2**	**0.3**	**0.4**

Supplementary Figure S 7: The maximum tolerable initial displacement of the sail with respect to the center of the beam and its associated maximum endurable initial tilt for having a stable propulsion for the case the dual pattern sail is driven by a (a) uni-modal and (b) multi-modal laser beam.

S5. Further Information on Lightsail Motion-trajectory

As discussed in the main manuscript, we have elaborated the real-nonlinear response of the spacecraft in the acceleration period using the Runge-Kutta method. It is noteworthy that we have obtained the motion-trajectory of the sail for an initial displacement and rotation separately in the main manuscript. However, it is a good practice to demonstrate the durability of the structure in the presence of both tilt and displacement as this may be the case in future lightsail deployments. We have limited our study to $\theta_{y_{max}} = 5°$ to comply well with the assumption of $\sin(\theta) = \theta$ of main manuscript. The plotted table in Supplementary Figure S8(a) depicts the maximum tolerable initial displacement and its associated maximum tolerable initial deviation for first design where a uni-modal beam is propelling a dual-pattern sail. The obtained robustness of the sail against relatively large displacements and rotations is a direct result of adding a transmissive layer and performing proper multi-objective optimization. As discussed comprehensively in the manuscript, addition of transmissive layer minimizes the cross-coupling between the rotation and displacement which leads to such a rigorous design. Next, we have performed similar analysis for the second design where a dual-patter metasail is being propelled by a multi-modal beam. In this case, the structure is less tolerable to larger initial displacements in comparison with first design, however remains stable for the reported initial conditions.

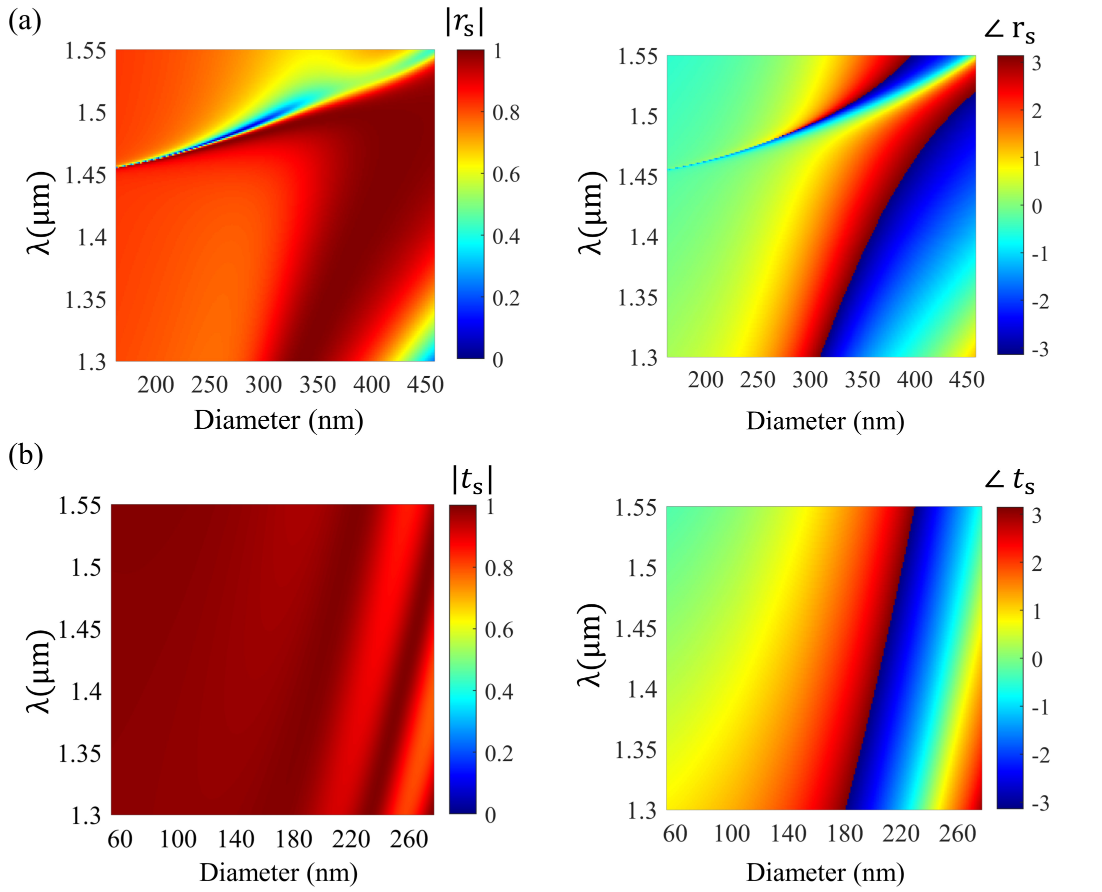

Supplementary Figure S 8: (a) The reflection amplitude and phase of the reflective unitcells under illumination of an s-polarized plane wave. (b)The transmission amplitude and phase of the transmissive unitcells under illumination of an s-polarized planewave.

S6. Effect of Increment in Loss on the Response of Elements

As discussed in the main manuscript, impacts of addition of loss to the dielectric elements of our structure can be studied by recalculating the optical response of the reflective and transmissive unitcells. Absorption coefficient of silicon is mainly governed by the defect-derived features which diminishes by increasing the operating wavelength. Furthermore, during our simulations in the main manuscript we have used the experimentally measured values of imaginary part of the silicon permittivity in the range of $\lambda = [1.3 - 1.45]$ μm that lies in the range of $\epsilon_{imag} = [10^{-8} - 10^{-12}]$[2]. To the best of authors knowledge, no experimental data is available for the remaining part of the propulsion spectrum. Hence we have extrapolated the value of the extinction coefficient for the rest of the propulsion band from the available data. Next, considering the uncertainly in the values of experimentally measured absorption coefficient, we have considered the imaginary part of the permittivity of the silicon as $\epsilon_{\text{image}} = 10^{-3}$[3]. It should be noted that we have selected this value for the case of study and may not be accurate and realistic. Also, the extinction coefficient of the silica is fixed as $\alpha_{SiO_2} = 10^{-6}$ based on the realistic values for loss in the near-infrared range[4]. The obtained results for both reflective and transmissive elements are depicted in Supplementary Figure S8(a,b). As can be seen, increasing the extinction coefficient of silicon and silica has minimal impact on the optical response of the dielectric unitcells which makes them robust against the uncertainties in the value of the extinction coefficient of the used materials. Nevertheless, enlargement in the values of the dielectric loss will increase the absorbed power while it is under illumination of a high-power laser beam which should be studied further. However, it has been proposed that by increasing the thickness of the silica layer which in turn increase the radiated power from the sail, can assist the temperature control of the nanocraft. The cost of such thickness enlargement will be total mass enhancement that extends the acceleration time

and distance.

References

[1] The Mathworks, Inc., Natick, Massachusetts, *MATLAB version 9.10.0.1613233 (R2021a)*, 2021.

[2] E. D. Palik, *Handbook of optical constants of solids*, vol. 3. Academic press, 1998.

[3] C. Schinke, P. Christian Peest, J. Schmidt, R. Brendel, K. Bothe, M. R. Vogt, I. Kröger, S. Winter, A. Schirmacher, S. Lim, *et al.*, "Uncertainty analysis for the coefficient of band-to-band absorption of crystalline silicon," *AIP Advances*, vol. 5, no. 6, p. 067168, 2015.

[4] O. Ilic, C. M. Went, and H. A. Atwater, "Nanophotonic heterostructures for efficient propulsion and radiative cooling of relativistic light sails," *Nano letters*, vol. 18, no. 9, pp. 5583–5589, 2018.
